# The Spatial Proximity of CD8^+^ FoxP3^+^PD-1^+^ Cells to Tumor Cells: A More Accurate Predictor of Immunotherapy Outcomes in Advanced Non-Small-Cell Lung Cancer

**DOI:** 10.3390/curroncol32050262

**Published:** 2025-04-30

**Authors:** Zijuan Hu, Zhihuang Hu, Keji Chen, Huixia Huang, Xinyang Zhong, Yaxian Wang, Jiayu Chen, Xuefeng He, Di Shi, Yupeng Zeng, Jiwei Li, Xiaoyan Zhou, Ping Wei

**Affiliations:** 1 Cancer Institute, Fudan University Shanghai Cancer Center, 270 Dong’an Road, Shanghai 200032, China; huzj23@m.fudan.edu.cn (Z.H.); 22211230011@m.fudan.edu.cn (K.C.); 21211230008@m.fudan.edu.cn (H.H.); 2 Department of Oncology, Shanghai Medical College, Fudan University, 270 Dong’an Road, Shanghai 200032, China; zhihuanghu@fudan.edu.cn (Z.H.); 20211230031@fudan.edu.cn (X.Z.); yaxianwang21@m.fudan.edu.cn (Y.W.); 22211230010@m.fudan.edu.cn (J.C.); 19211230006@fudan.edu.cn (X.H.); 19111230021@fudan.edu.cn (D.S.); 20111230053@fudan.edu.cn (Y.Z.); 3 Department of Pathology, Fudan University Shanghai Cancer Center, 270 Dong’an Road, Shanghai 200032, China; 4 Department of Thoracic Medical Oncology, Fudan University Shanghai Cancer Center, 270 Dong’an Road, Shanghai 200032, China; 5 Department of Colorectal Surgery, Fudan University Shanghai Cancer Center, 270 Dong’an Road, Shanghai 200032, China; 6 Institute of Pathology, Fudan University, 270 Dong’an Road, Shanghai 200032, China; 7 Department of Oncology, The Second Xiangya Hospital, Central South University, Changsha 410011, China; lijiwei2021@csu.edu.cn

**Keywords:** CD8^+^FoxP3^+^PD-1^+^ cells, spatial proximity, PD-1/PD-L1 blockade, therapeutic outcomes, advanced non-small-cell lung cancer

## Abstract

Background: To optimize precision immunotherapy for advanced NSCLC, comprehensive tumor immune microenvironment (TIME) characterization is crucial for efficacy prediction. Methods: Pretreatment tumor samples from 46 advanced NSCLC patients treated with PD-1/PD-L1 inhibitors were analyzed. The subregional abundance and spatial proximity scores of TIME cell subpopulations in 27 samples were assessed via multiplex immunohistochemistry (mIHC) targeting pan-CK, CD163, CD8, FoxP3, PD-1, and PD-L1. Correlations between the TIME features, clinicopathologic factors, treatment response, and prognosis were evaluated. Results: CD8^+^FoxP3^+^ cells were identified in NSCLC tissues, predominantly expressing PD-1/PD-L1. The PD-L1 TPS subgroups showed significant immune cell density/proximity differences, but CD8^+^FoxP3^+^PD-1^+^ infiltration was PD-L1 TPS-independent. Responders had higher CD8^+^FoxP3^+^PD-1^high^ density (*p* = 0.0497) and proximity scores (*p* = 0.0099) than non-responders. The CD8^+^FoxP3^+^PD-1^+^ presence and tumor proximity were essential for favorable outcomes. In low-PD-L1 TPS patients, the CD8^+^FoxP3^+^PD-1^+^ abundance and proximity scores strongly predicted the response (AUC: 0.79 and 0.75 vs. PD-L1 TPS AUC = 0.58). A survival analysis linked the presence and proximity score of CD8^+^FoxP3^+^PD-1^+^ cells to prolonged overall survival (OS) and progression-free survival (PFS). Notably, a low proximity score of CD8^+^FoxP3^+^PD-1^+^ cells emerged as an independent risk factor for a shorter PFS (HR = 6.16, 95% CI: 2.12–17.93, *p* = 0.001). Conclusion: The CD8^+^FoxP3^+^PD-1^+^ spatial proximity to tumor cells robustly predicts improved immunotherapy outcomes in advanced NSCLC.

## 1. Introduction

Lung cancer, particularly non-small-cell lung cancer (NSCLC), is a significant global health issue due to its high rates of morbidity and mortality [1,2,3]. And effectively intervening in advanced NSCLC has been a major concern in the field of lung cancer management [4]. With the immunological perspectives and techniques greatly serving the tumor etiology research in recent years, the tumor immune microenvironment (TIME) is recognized as a critical contributor to the malignant disease progression [5,6], and its complexity is increasingly revealed. Immune checkpoint blockade therapy represented by PD-1/PD-L1 inhibition is the most promising paradigm for the application of these concepts to therapeutic strategies [7,8], showing a favorable safety profile and reliable clinical benefit in advanced NSCLC patients [9,10,11,12].

However, therapeutic opportunities often present challenges, with individual differences in treatment response highlighting the need for precision therapy [13,14,15]. The current clinical decision support tools such as the immunohistochemical testing of the PD-L1 tumor proportion score (PD-L1 TPS) seem to be unsatisfactory in accurately screening the target population [16]. A deep understanding of the broader, more tangible TIME may provide an outlet for the solution. The TIME profiles of melanoma, gastric cancer, and lung cancer based on the co-labeling of multiple immune-related proteins have been revealed recently, and the phenotypic complexity and variable infiltration patterns of immunocytes provides visual evidence of the commonality and heterogeneity of the TIME between individuals [17,18,19,20]. Most surprisingly, the infiltration of certain immunocyte populations demonstrated remarkable predictive capacities for immunotherapy efficacy [17,18,19,21]. Besides the absolute amount of infiltrating immunocytes, the spatial location features of immunocytes were also shown to be potential biomarkers [18,20]. It has been pointed out that the personalized immunotherapy might require an analysis of the TIME cellular components in treatment baseline tumor biopsies [22].

Among the TIME, it is true that the exhaustion and activation of whole CD8^+^ T cells are of paramount importance, serving as the foundation of the rationale behind immune checkpoint inhibitor therapies. Nevertheless, the significance of various CD8^+^ T-cell subpopulations in the context of anti-tumor immunity deserves deeper investigation. The CD8^+^FoxP3^−^ subpopulation constitutes the majority and is the most prevalent, whereas the CD8^+^FoxP3^+^ subpopulation is less common and has historically been less scrutinized [19,23]. Studies have indicated that this latter group tends to exhibit a pronounced immune-suppressive profile, and the restoration of its anti-tumor capabilities may be crucial [19,24,25]. The role of CD8^+^FoxP3^+^ cells and their subsets in immunotherapy in advanced NSCLC, or their potential as predictive biomarkers, warrants further exploration.

In this study, we performed multiplex immunohistochemistry (mIHC) to simultaneously detect six TIME markers, including pan-CK, CD163, CD8, forkhead box P3 (FoxP3), PD-1, and PD-L1, for the in situ labeling and defining of tumor cells, T cells, and tumor-associated macrophages (TAMs), in pre-treatment tumor tissues from 46 advanced NSCLC patients who underwent PD-1/PD-L1 blockade therapy. Based on 27 high-quality stained samples, a panoramic view of the advanced NSCLC TIME was visualized, and a quantitative abundance and spatial analysis of the phenotypically defined cell subpopulations was conducted to comprehensively characterize the TIME. And we finally identified the presence and spatial proximity of CD8^+^FoxP3^+^PD-1^+^ cells as potential biomarkers to predict the treatment response and immunotherapy-related survival of advanced NSCLC patients.

## 2. Materials and Methods

### 2.1. Patients and Samples

A total of 46 patients with locally advanced or metastatic NSCLC who had undergone PD-1/PD-L1 blockade therapy were initially enrolled in this study. Pre-treatment tumor FFPE sections were collected for mIHC staining. The optimal immunotherapy efficacy of the patients was evaluated according to RECIST v1.1 criteria. Responders were defined as patients exhibiting complete response (CR) or partial response (PR), whereas non-responders comprised those with stable disease (SD) or progressive disease (PD). Baseline clinicopathologic characteristics, overall survival (OS; immunotherapy initiation to death), and progression-free survival (PFS; immunotherapy initiation to first progression/death) were systematically evaluated. All procedures, involving clinical data and archival biospecimens collected during routine care, were approved by the Ethics Committee of Fudan University Shanghai Cancer Center (No. 050432-4-1805C) and conducted in accordance with the Declaration of Helsinki. Written informed consent was obtained from all participants.

### 2.2. mIHC Staining and Multispectral Imaging

For each patient, one representative FFPE section was selected for mIHC analysis. The staining was performed using the following antibodies, targeting pan-CK (clone C-11, ab7753, Abcam, Cambridge, UK), CD163 (clone EPR19518, ab182422, Abcam), CD8 (clone SP239, ab178089, Abcam), FoxP3 (clone 236A/E7, ab20034, Abcam), PD-1 (clone EPR4877-2, ab137132, Abcam) and PD-L1 (clone SP142, ab228462, Abcam). Manual mIHC staining were conducted with the Opal Polaris 7-Color Manual IHC Kit (NEL861001KT, Akoya Biosciences, Marlborough, MA, USA), and all procedures were carried out under the guidance of the manual. Briefly, all sections were baked, dewaxed, rehydrated, and then subjected to antigenic repair. Next, the first primary antibody was applied to incubate the tissues, followed by Opal Polymer HRP incubation and Opal signal generation. Subsequently, microwave treatment was performed again for antibody stripping, and a new round of staining was turned on until all 6 markers were labeled. After the employment of spectral DAPI dye to stain the cell nuclei, the tissues were sealed with mounting medium against fluorescence quenching. Detailed staining conditions and steps are described in Table S1. The stained slides were scanned and visualized on the Vectra Polaris multispectral microscope system (Akoya Biosciences). All steps were batch-processed.

### 2.3. Image Data Extraction and Analysis

After quality assessment by two pathologists, 27 out of 46 cases underwent blind quantitative analysis on HALO software (India Labs, Tamil Nadu, India, version 3.2). DAPI-based cell segmentation was used to ensure cell phenotyping, counting, and localization analysis. Tissue was differentiated into the tumor parenchyma (tumor) and stoma based on cytokeratin-positive cell aggregation. PD-1/PD-L1-positive cells were further categorized into PD-1/PD-L1 high-, mid- and low-expression subgroups by fluorescence intensity tertile. The TIME cells were defined as 88 subpopulations of different phenotypes based on the expression and colocalization pattern of the six markers in a rational combination manner. The densities of cell subpopulations in the tumor, stroma, and overall region were calculated. Spatially, we calculated the average number of pan-CK^+^ tumor cells localized within 30 μm of immune cell nuclei and defined it as the proximity score of immune cells, which was driven by the well-established biological and clinical relevance of immune tumor cell interactions within a 30 μm radius and the consideration to maintain cross-study consistency [18,20,26].

### 2.4. Statistics

Subregional cell densities and proximity scores were compared among patient groups using Mann-Whitney U or Kruskal-Wallis tests with Benjamini-Hochberg correction for multiple comparisons. Survival was analyzed using Kaplan-Meier curves and log-rank tests. Prognosis predictors were determined through univariate and multivariate Cox regression analysis. The performance of prediction signature was measured by the area under the curve (AUC). Data processing was conducted on the SPSS (IBM, Armonk, NY, USA, version 26.0) or R software (version 4.0.3) [27]. All *p*-values were two-sided.

## 3. Results

### 3.1. Clinicopathologic Features of NSCLC Patients

**Figure 1:**
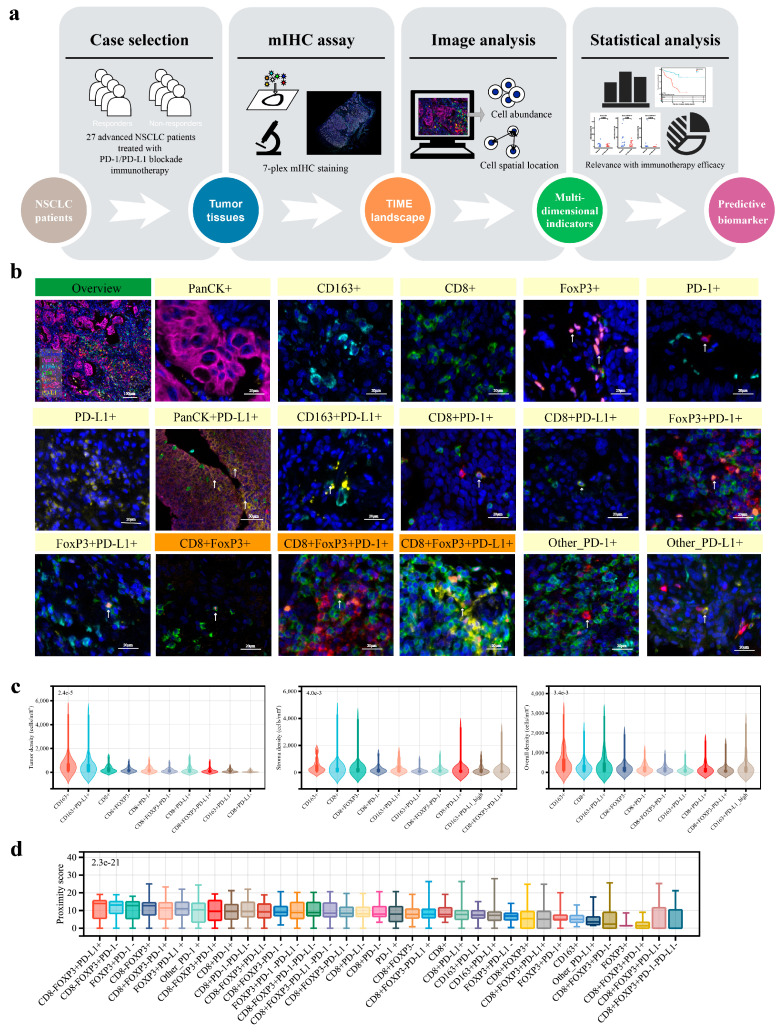
Overview of the TIME of advanced NSCLC: (**a**) diagram summarizing the study design; (**b**) representative mIHC images of the overall TIME, and the cell phenotypes of all possible copositive combinations of the 6 targets, as well as the subsets of other_PD-1+ and other_PD-L1+; (**c**) immunocyte subpopulations that are ranked in the top 10 based on median density in the tumor area, stroma area, and whole slide; and (**d**) immunocyte subpopulations sorted according to the median proximity score.

Pre-treatment samples were collected from 46 advanced NSCLC patients who had undergone PD-1/PD-L1 blockade therapy, and 27 cases were finally selected for a quantitative image analysis of the tumor immune microenvironment (TIME). The flow chart of case selection and experimental design are shown in Figure 1a and Figure S1. The baseline clinicopathologic features and immunotherapy profiles of the 27 patients are present in Table 1 and Table S2.

In short, the median age of the patients was 58; 63% (17/27) were male. PD-L1 TPS assays were performed and 19 cases had a PD-L1 TPS > 1%, including 8 with a PD-L1 TPS ≥ 50%. The timing of patients receiving immunotherapy ranged from first-line to third-line treatment; the treatment regimens included anti-PD-1/PD-L1 monotherapy (16/27), PD-1/PD-L1 and CTLA-4 dual-blockade therapy (3/27), and PD-1/PD-L1 inhibition combined with chemotherapy (8/27). For the objective response, 11 patients achieved PR, 4 were assessed for SD, and the rest were assessed for PD.

### 3.2. Analysis Overview of the Advanced NSCLC TIME

The TIME landscape of the 27 specimens was visualized after mIHC staining and multispectral imaging. Representative fluorescence images of each target and a multifluorescence fused landscape are shown in Figure S2a. For all mIHC images, we completed a basic cell segmentation and positive signal interpretation (Figure S2b) and categorized the tumor tissue into tumor and stroma subregions based on cytokeratin-positive cell aggregation (Figure S2c,d). We defined 88 cell populations based on the potential co-expression patterns of CD8, CD163, FoxP3, PD-1, PD-L1 and pan-CK, as well as their potential clinical significance, ultimately (Tables S3 and S4, Figure 1b and Figure S2e). For each immunocyte phenotype, we enumerated and determined the density within both the tumor and stromal regions. Furthermore, to evaluate the spatial proximity between these immune cells and tumor cells, we calculated the mean count of tumor cells within 30 μm around the nucleus of the certain immunocyte subset as its proximity score (Figure S2f,g).

The TIME characteristics were preliminarily quantified by the measurement of the subregional abundance of immunocyte subpopulations. CD163^+^ and CD8^+^ cells were frequent in both tumor and stroma, while the FoxP3^+^ cell density was relatively low (Figure S3a–c). When comparing the tumor region with the stroma region, there are differences in the abundance structure of immune cell subsets. Inside the tumor parenchyma, the CD163^+^PD-L1^+^ cells were the most densely populated subset. In the stroma, the most dominant immunocyte subpopulation was CD8^+^FoxP3^−^ cells (Figure 1c). Notably, CD8^+^FoxP3^+^ cells, which were considered a rare immunocyte subpopulation, were observed in advanced NSCLC tissue (Figure 1b). Moreover, there were CD8^+^FoxP3^+^ cell subsets that prominently expressed PD-1 or PD-L1 proteins, indicating a potential phenotype of T-cell exhaustion or immune suppression in these cells. For each immune cell subset, we did not find significant differences in their abundance between the tumor parenchyma and the stromal regions (Figure S4).

Examining the spatial positioning of immune cells relative to tumor cells further elucidates the TIME characteristics. The proximity scores of these immunocyte subpopulations showed significant variation among individuals. And, when comparing different immune cells, several subsets of FoxP3^+^ cells ranked at the forefront in terms of median proximity scores (Figure 1d). We specifically noted that CD8^+^FoxP3^+^ cells and their subsets were within 30 μm of a significant number of tumor cells. This indicated that the spatial interactions between them and tumor cells should be taken seriously.

Altogether, the TIME of advanced NSCLC is complex and exhibits significant heterogeneity among individuals. The indicative value of the presence and spatial characteristics of CD8^+^FoxP3^+^ cells may require further investigation.

### 3.3. The CD8^+^FoxP3^+^ Cells Exhibited Distinctive Correlations with Clinicopathological Parameters and Expression Profiles of PD-1/PD-L1

**Figure 2:**
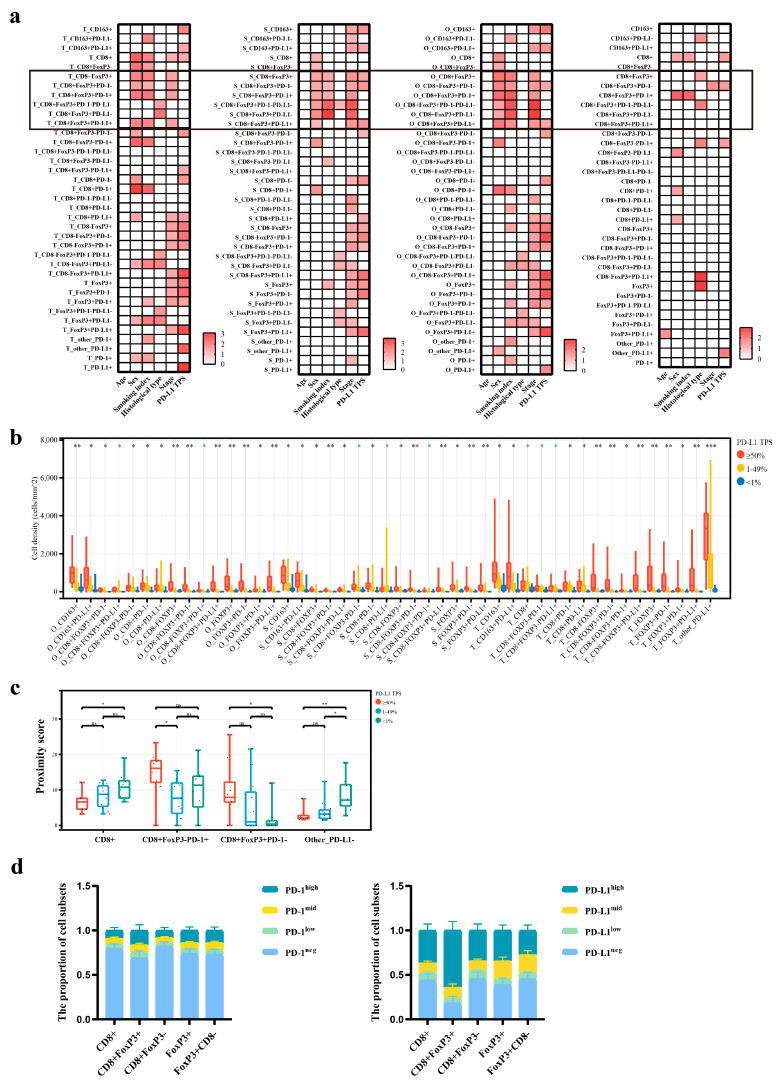
The correlation of CD8^+^FoxP3^+^ cells with clinicopathologic traits and their PD-1/PD-L1 expression features: (**a**) significant associations of immune cell subregion densities and proximity scores with clinicopathologic features, displaying -log_10_P-values for *p* < 0.05 (from left to right: tumor area density, stromal area density, overall density, and proximity score); (**b**) densities of immune cell subsets showing significant variation among PD-L1 TPS subgroups (* *p* < 0.05, ** *p* < 0.01, *** *p* < 0.001, and the color black indicated significant Kruskal–Wallis test *p*-values for the three groups, while green showed significant *p*-values from pairwise Dunn’s Test despite non-significant overall differences); (**c**) proximity scores of immune cell subsets showing significant variation among PD-L1 TPS subgroups (* *p* < 0.05, and ** *p* < 0.01); and (**d**) comparison of proportions of T-cell subsets with different PD-1/PD-L1 levels.

Subsequently, we conducted an exploratory assessment of immune cell abundance dynamics across clinicopathological subgroups. We hypothesized that CD8^+^FoxP3^+^ cells were the most frequently varying immune cell population, with the density of the overall population or relevant subsets significantly correlated with sex, smoking history, histological type, disease stage, and PD-L1 TPS (Figure 2a). In brief, tissues from male patients and heavy smokers contained a higher abundance of CD8^+^FoxP3^+^ cells; compared to adenocarcinomas, the density of CD8^+^FoxP3^+^PD-L1^−^ cells was higher in the tumor areas of non-adenocarcinomas; and, compared to stage IV tumors, the density of CD8^+^FoxP3^+^ cells was higher in stage IIIB/IIIC tumors (Figure S5). It is currently recognized that tumor cell PD-L1 expression correlates with the infiltration of immune cells [28,29,30,31]. In our study, samples with a high PD-L1 TPS exhibited significantly higher levels of various immune cells (Figure 2b). However, the CD8^+^FoxP3^+^ subpopulation was relatively less impacted, and we observed no correlation between PD-L1 TPS and the stromal, tumor, or overall density of the specific CD8^+^FoxP3^+^PD-1^+^ subset (Figure 2a).

Then, we explored potential links between immune-tumor spatial proximity (a key TIME feature) and clinicopathological factors. Notably, the CD8^+^FoxP3^+^ cell subpopulation exhibited dynamic heterogeneity and retained distinct phenotypic features. For example, the proximity score of CD8^+^FoxP3^+^PD-1^+^ cells was higher in male tissues than in female tissues; patients who smoked more showed a higher proximity score of CD8^+^FoxP3^+^PD-1^+^ subsets; the proximity score of CD8^+^FoxP3^+^PD-L1^−^ cells was lower in adenocarcinoma than in non-adenocarcinoma tissues: and, comparing tumors of stage IIIB/IIIC to those of stage IV, the proximity score of CD8^+^FoxP3^+^PD-1^−^ cells was higher (Figure S6). And the PD-L1 TPS potentially affected the degree of proximity of specific T cells to tumor cells. It seemed that tumors with an elevated PD-L1 expression decreased the engagement with the general CD8^+^ T-cell pool but increased interactions with subsets like CD8^+^FoxP3^−^PD-1^+^ and CD8^+^FoxP3^+^PD-1^−^ (Figure 2c). Most importantly, we found that the proximity score of CD8^+^FoxP3^+^PD-1^+^ cells was unrelated to PD-L1 TPS levels (Figure 2a), which was consistent with the results regarding cell abundance mentioned earlier.

Next, we investigated the PD-1/PD-L1 expression patterns in CD8^+^FoxP3^+^ cells. We found that the majority of CD8^+^FoxP3^+^ cells exhibited the expression of either PD-1 or PD-L1, with a notably increased proportion of high subgroups, while the global CD8^+^ cells and CD8^+^FoxP3^−^ subsets, as well as the global FoxP3^+^ cells and FoxP3^+^CD8^−^ subsets, respectively, shared similar profiles in immune checkpoint expression (Figure 2d). Consequently, CD8^+^FoxP3^+^ cells might have a specialized role and occupy a unique niche in the context of T-cell exhaustion and tumor immune modulation.

Taken together, our data suggested that the majority of CD8^+^FoxP3^+^ cells expressed either PD-1 or PD-L1, and the expression levels were relatively high. Meanwhile, the CD8^+^FoxP3^+^PD-1^+^ cells attracted our attention as a rare subpopulation of TIME cells with variants independent of PD-L1 TPS in terms of both abundance and spatial location, which could potentially suggest its unique advantage as a predictive marker for immunotherapy. It should be noted, however, that the subgroup analyses—limited by the cohort size—were exploratory in nature, and these findings warrant validation in larger cohorts and across diverse analytical frameworks.

### 3.4. The Abundance and Spatial Proximity of CD8^+^FoxP3^+^PD-1^+^ Cells Provided Superior Predictive Efficacy for Immunotherapy than PD-L1 TPS

**Figure 3:**
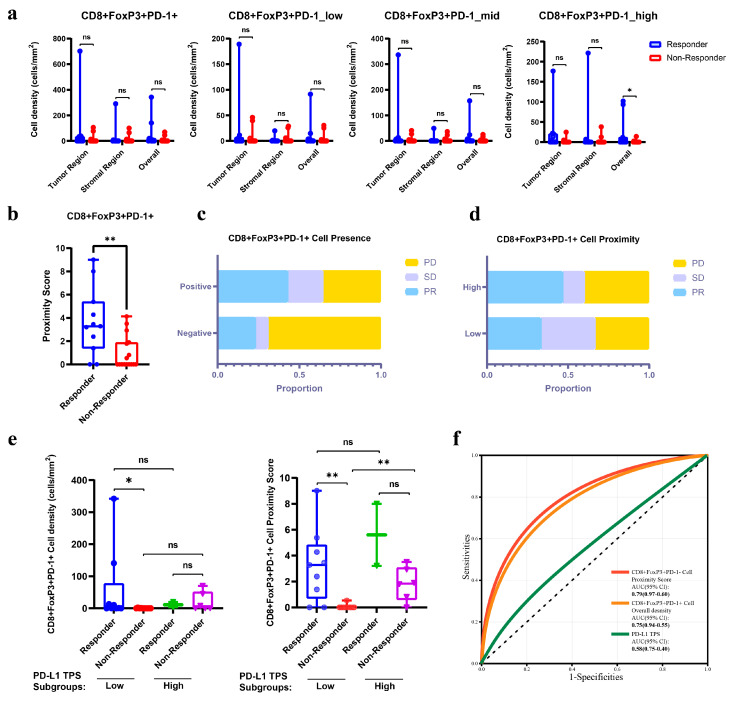
The predictive role of CD8^+^FoxP3^+^PD-1^+^ cells in response to PD-1/PD-L1 inhibitors: (**a**) variations in the subregional abundance of CD8^+^FoxP3^+^PD-1^+^ cell subsets between immunotherapy responders and non-responders; (**b**) variations in the proximity score of CD8^+^FoxP3^+^PD-1^+^ cells between immunotherapy responders and non-responders; (**c**) comparison of immunotherapy efficacy between subgroups of patients with present and absent CD8^+^FoxP3^+^PD-1^+^ cells in tissues; (**d**) comparison of immunotherapy efficacy between subgroups of patients with proximity-high and -low CD8^+^FoxP3^+^PD-1^+^ cells; (**e**) differentials in the density and proximity score of CD8^+^FoxP3^+^PD-1^+^ cells in PD-L1 TPS subgroups between responders and non-responders (with a PD-L1 TPS cutoff value of 50%); (**f**) ROC curves for predicting objective response to immunotherapy using CD8^+^FoxP3^+^PD-1^+^-cell proximity score, density, and PD-L1 TPS. (* *p* < 0.05, and ** *p* < 0.01).

To explore the potential and relevance of CD8^+^FoxP3^+^PD-1^+^ cells as predictive markers of immunotherapy efficacy, we performed a differential analysis of the abundance and spatial characteristics of TIME cell subpopulations between the immunotherapy-responsive and non-responsive groups. Our results showed that, compared to non-responders, responders had a tendency towards higher densities of CD8^+^FoxP3^+^PD-1^+^ cells, with CD8^+^FoxP3^+^PD-1^high^ showing a statistically significant difference in overall density between the two (Figure 3a). Spatially, the proximity scores of CD8^+^FoxP3^+^PD-1^+^ cells in responder tissues were also significantly higher than in non-responders (Figure 3b). We further examined the impact of the presence of CD8^+^FoxP3^+^PD-1^+^ cells in tissues on patients’ objective responses. We found that the positive group (with objective CD8^+^FoxP3^+^PD-1^+^ cells in their tissues) had a significantly higher rate of PR and SD outcomes, along with a markedly reduced rate of PD (Figure 3c). For patients with present CD8^+^FoxP3^+^PD-1^+^ cells, we divided them into two groups based on the presence or absence of tumor cells within 30 μm: proximity-high and -low. We observed that the proportion of patients achieving PR was also higher in the high group compared to the low group (Figure 3d).

Given that the increased presence and spatial proximity of CD8^+^FoxP3^+^PD-1^+^ cells appeared to indicate improved treatment efficacy, the question arose whether this predictive significance retained irreplaceable value when considered within the broader context of the TIME. In terms of quantity, a total of 53 indicators of subregional cell density varied significantly between tissues from responders and non-responders (*p* < 0.05, Table S5, Figure S7). In a nutshell, more abundant CD8^+^ cells in either the tumor or stroma were associated with a positive treatment response, especially the CD8^+^PD-1^+^ and the CD8^+^FoxP3^−^ subsets. Meanwhile, higher tumoral and stromal densities of CD163^+^PD-L1^+^ immunocytes, especially the CD163^+^PD-L1^high^ subpopulation, were observed in non-responder samples (Table S5, Figure S7). And, spatially, the proximity scores of three subsets had predictive value of the treatment response (*p* < 0.05, Table S6 and Figure S8): higher proximity scores of CD8^+^FoxP3^+^PD-1^+^ and CD8^+^PD-1^+^ immunocytes appeared in responders, while CD8^+^PD-L1^−^ cells behaved in the opposite way. Apparently, in both abundance and spatial proximity, the CD8^+^FoxP3^+^PD-1^+^ cell subpopulation held up as a robust and significant positive predictor for immunotherapeutic efficacy, which was remarkable in the entire TIME immune cell population. The distribution of densities and proximity scores of all TIME cell subsets across different clinical pathological factors and treatment response subgroups is shown in Figure S9.

Our data also indicated that the predictive power of PD-L1 expression levels on tumor cells for immunotherapy efficacy was compromised, with no significant differences in the abundance of pan-CK^+^PD-L1^+^ cells between responders and non-responders (Table S5). This also indirectly illustrates the predictive power of PD-L1 TPS, as the abundance of pan-CK^+^PD-L1^+^ cells detected by mIHC is consistent with the clinical PD-L1 TPS (Figure S5e). However, a subgroup difference analysis showed that, in the subgroup, with low PD-L1 TPS levels, a higher abundance and proximity score of CD8^+^FoxP3^+^PD-1^+^ cells were both enriched in responders, although similar significant findings were not observed in the subgroup with a high PD-L1 TPS due to limitations in the cohort size (Figure 3e). This finding indicated that the level of CD8^+^FoxP3^+^PD-1^+^ cell infiltration could help identify responders within the low PD-L1 TPS patient group. The ROC curve also highlighted that the proximity score and density of CD8^+^FoxP3^+^PD-1^+^ cells served as superior predictive markers for treatment efficacy compared to PD-L1 TPS (AUCs: 0.79 vs 0.75 vs 0.58, Figure 3f).

### 3.5. The Proximity Score of CD8^+^FoxP3^+^PD-1^+^ Cells Was an Independent Prognostic Factor for Immunotherapy-Related PFS in Advanced NSCLC

To uncover the predictive value and significance of the CD8^+^FoxP3^+^PD-1^+^ cell infiltration for post-immunotherapy survival, we performed an analysis correlating all TIME features with patients’ prognosis. Regarding cellular abundance, high-density CD8^+^ cells (mainly FoxP3- or PD-1-negative subsets) were indicative of better overall survival (OS), while several subsets of FoxP3^+^PD-L1^+^ in the tumor area suggested a poorer prognosis (Figure S10a and Table S7). And the progression-free survival (PFS) period was linked to similar cell subpopulations, with the density of FoxP3^+^PD-L1^+^ cells, especially CD8^−^FoxP3^+^PD-L1^+^ cells, in both the tumor and stromal area being a risk factor, and the density of CD8^+^FoxP3^−^ cells emerging as the most significant protective factor (Figure S10b and Table S8). We noted that the subregional density of CD8^+^FoxP3^+^PD-1^+^ cells and their subsets did not correlate with either OS rates or PFS rates (Tables S7 and S8). Given the relatively low prevalence of CD8^+^FoxP3^+^PD-1^+^ cells in samples and their non-dominant status among cellular subsets, we explored the prognostic implications of its presence in tissues. Meaningfully, the presence of CD8^+^FoxP3^+^PD-1^+^ cells was found to be predictive of extended OS and PFS (Figure S10c,d).

**Figure 4:**
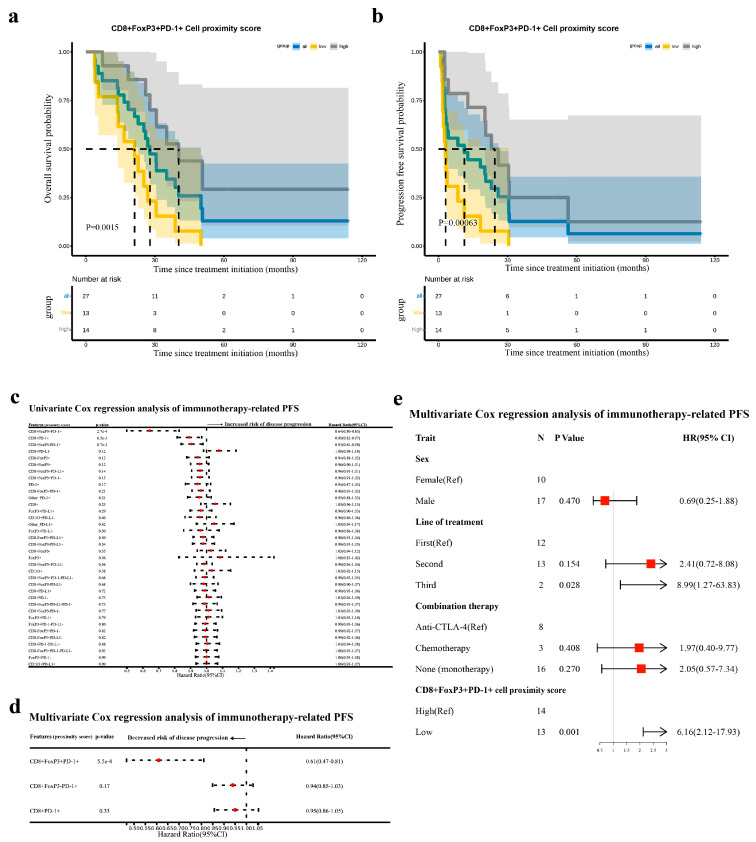
The prognostic value of the proximity score of CD8^+^FoxP3^+^PD-1^+^ cells in immunotherapy in advanced NSCLC: (**a**) survival analysis of the proximity score of CD8^+^FoxP3^+^PD-1^+^ cells for OS (high: patients with a high proximity score; low: patients with a low proximity score; patients were grouped by the median value); (**b**) survival analysis of the proximity score of CD8^+^FoxP3^+^PD-1^+^ cells for PFS (high: patients with a high proximity score; low: patients with a low proximity score; patients were grouped by the median value); (**c**) forest plot of the results from the univariate Cox regression analysis for immunocyte proximity scores associated with OS; (**d**) forest plot of the results from the multivariate COX regression analysis of immunocyte proximity scores significant in univariate analysis; and (**e**) forest plot of the results from the multivariate COX regression analysis of PFS considering clinicopathological characteristics and immunotherapeutic regimens (only covariates demonstrating *p* < 0.1 in univariate analyses were included; patient subgroups with high and low CD8^+^FoxP3^+^PD-1^+^-cell proximity score were defined by the median value).

Subsequently, we investigated whether the spatial positioning of immune cells relative to tumor cells correlates with the prognosis. To our surprise, the proximity score of CD8^+^FoxP3^+^PD-1^+^ cells was a predictive marker for both OS and PFS, with a higher proximity score indicating a reduced risk of mortality and disease progression (Figure 4a–c and Figure S11a, Tables S9 and S10). When accounting for various spatial relationships between immune and tumor cells in the multivariate analysis, CD8^+^FoxP3^+^PD-1^+^ cells did not independently correlate with OS (Figure S11b), but its predictive power for PFS remained robust (Figure 4d). In addition, despite the presence of numerous confounding factors, such as diverse clinicopathological characteristics and immunotherapeutic regimens among patients (Figure S12), the proximity score of CD8^+^FoxP3^+^PD-1^+^ cells remained a trustworthy and independent predictor of PFS associated with immunotherapy (HR = 6.16, 95% CI: 2.12–17.93, *p* = 0.001, Figure 4e).

In summary, the presence of CD8^+^FoxP3^+^PD-1^+^ cells in advanced NSCLC tissues and their close spatial proximity to tumor cells might be crucial for favorable survival outcomes in patients receiving immunotherapy. And we identified the proximity score of CD8^+^FoxP3^+^PD-1^+^ cells as a novel and powerful biomarker for predicting PFS.

## 4. Discussion and Conclusions

The PD-1/PD-L1 blockade is a key strategy in advanced NSCLC management, but its efficacy is inconsistent due to inadequate patient selection. While PD-L1 TPS is used in treatment decisions, its predictive power is limited as some patients with a high tumor PD-L1 expression do not respond to immunotherapy, and others with a low expression do [16]. Other biomarkers like microsatellite instability (MSI) and deficient mismatch-repair (dMMR), tumor mutation burden (TMB), and Epstein–Barr virus (EBV) infection status have shown varied success in predicting treatment outcomes and have particularly uncertain application prospects in advanced NSCLC [17,32]. As tumor immunology and image analysis technology progress, the abundance and even the spatial positioning of immune cell subsets have become highly accessible, with the latter emerging as a novel yet significant dimension in the assessment of TIME [20,33,34]. For example, the average proximity of tumor cells to immunocytes within a defined spatial range is a key indicator, reflecting the spatial impact of immunocytes on tumor cells and serving as a promising predictor for patient survival and immunotherapy outcomes [18,20]. This study posits that exploring more realistic and specific TIME features might provide a way to better explain individual differences in immunotherapy efficacy, and helps to discover the exact TIME factors that predict therapeutic activity to guide patient stratification.

We took the exploration of the composition of important immunocyte subpopulations represented by T cells and TAMs as an entry point to glimpse the holistic picture of the advanced NSCLC TIME. We performed a fine phenotypic delineation of key immunocytes based on the targeted detection of CD163, CD8, FoxP3, PD-1, and PD-L1, and then defined their infiltration characteristics by counting regionally and calculating spatial proximity scores. In particular, given that T cells presented a stratified state of functional exhaustion at different PD-1 levels [19,22], we refined the TIME cells into distinct populations by the signal intensity of PD-1/PD-L1. Thus, we finally obtained a TIME characterization dataset containing information on marker intensity, multiple marker colocalization profiles, cell subregional abundance, and spatial proximity at the single-cell resolution level. We identified rare phenotypes, and CD8^+^FoxP3^+^ cells, as well as its subsets, which required further exploration for a thorough understanding of the TIME. Interestingly, compared with some important immune cells such as CD163^+^ cells, the CD8^+^FoxP3^+^ subset, which was not dominant in terms of abundance, exhibited a proximity score that was not low and should not be overlooked.

Although the functionality of the predominant CD8 and FoxP3-positive T-cell populations has been extensively studied, the characteristics of the subpopulation co-expressing both markers and their implications for immune checkpoint inhibitors have not garnered attention. A handful of studies have employed mIHC to identify the CD8^+^FoxP3^+^ cell subpopulation in advanced melanoma, ovarian cancer, and NSCLC, with emerging evidence suggesting that CD8^+^FoxP3^+^ cells may be a beneficial factor in the efficacy of immune checkpoint inhibitors [19,23,24,25]. In our samples, CD8^+^FoxP3^+^ cells were not the dominant T-cell subpopulation; yet, their overall or subpopulation abundance and proximity scores exhibited significant variation across various clinical pathological subgroups. This suggested that this cell population was dynamically active under different pathological factors and during disease progression. And we found that the variation in the abundance of CD8-, CD163-, and FoxP3-positive cell subpopulations within the PD-L1 TPS subgroups might be codirectional, with higher tumor PD-L1 levels associating with more diverse immune cell infiltration. Previous studies and our subsequent data also demonstrated that these immune cells had varying effects on the efficacy of immune therapy, which partially explained the low predictive power of PD-L1 TPS as a prognostic marker [17,18,29,31]. However, the abundance and density of the CD8^+^FoxP3^+^PD-1^+^ subpopulation did not show a significant correlation with PD-L1 TPS, suggesting its potential to serve as an independent, potentially more effective predictive factor in immune therapy outcomes. Furthermore, we found that CD8^+^FoxP3^+^ cells exhibited a high PD-1 and PD-L1 expression, distinguishing them from typical CD8^+^FoxP3^−^ or FoxP3^+^CD8^−^ cells. This aligned with the situation in ovarian cancer where the CD8^+^FoxP3^+^CD25^+^ T-cell subpopulation displayed high-level immune-suppressive characteristics phenotypically [25].

Our intergroup difference analysis revealed that the CD8^+^FoxP3^+^PD-1^+^ subpopulation clearly demonstrated a correlation with the immunotherapy response, and the trend was more pronounced in patients with low PD-L1 TPS levels. The presence of the CD8^+^FoxP3^+^PD-1^+^-cell subpopulation and its spatial proximity score both exhibited superior predictive efficacy compared to PD-L1 TPS. Notably, the proximity score of CD8^+^FoxP3^+^PD-1^+^ for PFS prediction was irreplaceable and independent, showing a promising prospect for clinical translation. Several studies have corroborated our findings and provided insights into the possible mechanisms [24,25]. In ovarian cancer, clinical samples and in vitro experiments suggested that elevated levels of CD8^+^FoxP3^+^CD25^+^ T cells were associated with a better efficacy of the PD-1 blockade. These cells were reported to exhibit an early effector memory phenotype with particularly high levels of PD-1, CTLA-4, TIM-3, and LAG-3 checkpoints, indicating long-term antigen stimulation. However, compared with the CD8^+^FoxP3^−^ subpopulation, CD8^+^FoxP3^+^ cells produced higher levels of Granzyme-B and effector cytokines, and they showed a higher proliferation capacity after polyclonal activation, suggesting a higher potential to enhance anti-tumor immunity and possibly being key executors of the anti-tumor immune response following the PD-1 blockade [25]. In patients with resectable NSCLC treated with neoadjuvant anti-PD-1, the density of CD8^+^FoxP3^+^ T cells in patients who achieved a major pathological response (MPR) was significantly increased, with the strongest association in PD-1-positive and PD-L1-negative subpopulations. Then, the single-cell RNA sequencing of the CD8^+^FoxP3^+^ T-cell subpopulation also revealed a highly activated and cytotoxic phenotype [24]. Our results further confirmed that, in advanced NSCLC, CD8^+^FoxP3^+^ T cells likely represented a group of T cells with special phenotypes and pivotal functions in immune responses. The CD8^+^FoxP3^+^PD-1^+^ cell group might retain the potential to perform effector functions, and become activated post PD-1 blockade, and its high proximity to tumor cells was conducive to maximizing its tumor-killing capabilities, potentially delaying disease progression and even achieving remission.

Compared to existing immunotherapy biomarkers, the CD8^+^FoxP3^+^PD-1^+^ cell proximity score may offer promising advantages in both methodological implementation and clinical significance, and could be a valuable adjunctive diagnostic tool for clinical therapeutic decision-making. A meta-analysis found that the mIHC-based biomarker strategy assessing the TIME demonstrated the best predictive efficacy, outperforming the PD-L1 TPS, TMB, and gene expression profiling (GEP) assessments [21]. And, for NSCLC immunotherapy, the utility of MSI, dMMR, and TMB remains particularly uncertain due to their low prevalence in NSCLC (<1% for MSI/dMMR) [35] and variable predictive performance (e.g., TMB cutoffs lack standardization) [32]. Notably, the CD8^+^FoxP3^+^PD-1^+^ cell proximity score demonstrated a superior predictive performance to PD-L1 TPS, the most widely validated and clinically actionable biomarker, particularly in identifying immunotherapy-responsive subgroups among patients with a low PD-L1 TPS. Critically, this spatial biomarker can be derived from a single FFPE slide using mIHC, offering cost-effectiveness and practical advantages over TMB or MSI/dMMR (which requires next-generation sequencing). Even though other molecular testing data were unavailable in our study and direct comparative analyses were lacking, our findings still posit that the CD8^+^FoxP3^+^PD-1^+^ cell proximity score may complement, refine, or even replace PD-L1-based decision-making. Our future validation studies aim to translate this discovery into clinical practice.

However, this study has several limitations. First, due to the current technical constraints of mIHC, our analysis could not comprehensively evaluate other critical TIME components, including B cells, myeloid-derived suppressor cells, dendritic cell subsets, and additional immune checkpoints/cytokines. Second, the cohort size was insufficient to establish a negative control group or an independent validation cohort, and the exclusion of 19/46 cases due to technical artifacts further reduced the analytical sample size. Although the retained cases preserved the original cohort’s clinical and biological diversity, a further study with an expanded sample size is necessary in order to validate the current findings. Third, we were unable to incorporate other established biomarkers of immunotherapy response (e.g., TMB and MSI/dMMR) due to insufficient tissue availability and retrospective data limitations, which may have omitted important confounding variables. Fourth, while spatial proximity metrics showed predictive value, the mechanistic basis of this association remains unexplored. Future studies integrating single-cell RNA sequencing, spatial transcriptomics, and in vitro/in vivo models could further validate these findings by delineating cellular phenotypes, spatial interaction networks, and molecular drivers of the CD8^+^FoxP3^+^PD-1^+^ tumor cell spatial relationship.

In summary, we have explored quite realistic and specific TIME features of advanced NSCLC by comprehensively assessing the infiltration abundance and spatial location of fine subpopulations of T lymphocytes and macrophages based on the combinatorial labeling of several key proteins. And we identify the proximity score of CD8^+^FoxP3^+^PD-1^+^ cells as an exact indicator that correlates with the efficacy of PD-1/PD-L1 blockade therapy and patients’ outcomes, which is encouraging and warranted further validation in larger clinical cohorts. In the future, we expect to expand the cohort size, integrate multi-omics profiling (including MSI/TMB), and prospectively validate spatial metrics against these benchmarks, and to employ single-cell or spatial transcriptome sequencing technologies and in vitro and in vivo experiments to provide mechanistic explanations for our findings.

## Figures and Tables

**Table 1:** Baseline clinicopathologic features and immunotherapy profiles of advanced NSCLC patients.

Items	Total N = 27
**Age**	
Median, IQR	58 (50,65)
**Sex**	
Male	17 (63.0%)
Female	10 (37.0%)
**Smoking history**	
≥400 cigarette-years	11 (40.7%)
<400 cigarette-years	16 (59.3%)
**ECOG PS**	
1	27 (100.0%)
**Disease stage**	
IIIB/IIIC	3 (11.1%)
IV	24 (88.9%)
**Histological type**	
Adenocarcinoma	13 (48.1%)
Non-adenocarcinoma ^a^	14 (51.9%)
**PD-L1 TPS**	
<1%	8 (29.6%)
≥1%	19 (73.4%)
1–49%	11 (40.7%)
≥50%	8 (29.6%)
**Line of treatment**	
First-line	12 (44.4%)
Second-line	13 (48.1%)
Third-line	2 (7.4%)
**Combination therapy**	
None (monotherapy)	16 (59.3%)
Anti-CTLA-4	3 (11.1%)
Chemotherapy	8 (29.6%)
**Best Response**	
CR	0 (0.0%)
PR	11 (40.7%)
SD	4 (14.8%)
PD	12 (44.4%)

^a^ This category includes squamous cell carcinoma (*n* = 12), adenosquamous carcinoma (*n* = 1), and sarcomatoid carcinoma (*n* = 1) in our cohort.

## Data Availability

The data used and/or analyzed during the current study are available from the corresponding author upon reasonable request.

## Supplementary Materials

The following supporting information can be downloaded at: https://www.mdpi.com/article/10.3390/curroncol32050262/s1, Figure S1: Flow chart of case selection and experimental design; Figure S2: Overview of the image analysis method; Figure S3: Violin plot of tumoral density, stromal density, and overall density of the immunocyte subpopulations; Figure S4: The results of the analysis of differences in the subregional abundance of each immunocyte subpopulation; Figure S5: Correlation between the densities of TIME cell subpopulations and clinicopathological factors; Figure S6: Correlation between the proximity scores of immunocyte subpopulations and clinicopathological factors; Figure S7: Results of the analysis of intergroup differences in cell subpopulation densities between responders and non-responders; Figure S8: Significant results of the analysis of intergroup differences in immunocyte proximity scores between responders and non-responders; Figure S9: An overview of the distribution of TIME features in the population within the advanced NSCLC cohort; Figure S10: Predictive value of densities of immunocyte subpopulations for immunotherapy-related survival; Figure S11: Predictive value of proximity scores of immunocyte subpopulations for immunotherapy-related OS; Figure S12: Forest plot of the results from the univariate Cox regression analysis of PFS including clinicopathological characteristics, immunotherapeutic regimens, and CD8^+^FoxP3^+^PD-1^+^ cell proximity score (patients were grouped by the median proximity score); Table S1: Experimental protocol for mIHC staining; Table S2: Clinicopathologic features and survival outcomes for individual patients; Table S3: Basic cell phenotypes for subpopulation analysis; Table S4: Cell phenotype definition associated with differential PD-1/PD-L1 expression levels; Table S5: Results of difference analysis of tumor TIME cell subpopulation densities between responders and non-responders; Table S6: Results of difference analysis of proximity scores of immunocyte subpopulations between responders and non-responders; Table S7: Univariate Cox regression analysis to assess the correlation between the cell subpopulation densities and immunotherapy-related OS; Table S8: Univariate Cox regression analysis to assess the correlation between the cell subpopulation densities and immunotherapy-related PFS; Table S9: Univariate Cox regression analysis to assess the correlation between the proximity scores of immunocyte subpopulations and immunotherapy-related OS; Table S10: Univariate Cox regression analysis to assess the correlation between the proximity scores of immunocyte subpopulations and immunotherapy-related PFS.
