# Supplementary material for: The Spatial Proximity of CD8+ FoxP3+PD-1+ Cells to Tumor Cells: A More Accurate Predictor of Immunotherapy Outcomes in Advanced Non-Small-Cell Lung Cancer
Source: Curr Oncol. 2025 Apr 30;32(5):262. doi: 10.3390/curroncol32050262 (PMC12110189; doi:10.3390/curroncol32050262)
Supplement: Supplementary file 1 [file curroncol-32-00262-s001.zip › curroncol-3544324-supplementary.pdf]

Supplementary Materials for

The Spatial Proximity of CD8$^+$FoxP3$^+$PD-1$^+$ Cells to Tumor Cells: A More Accurate Predictor of Immunotherapy Outcomes in Advanced Non-small-Cell Lung Cancer

Zijuan Hu [†], Zhihuang Hu [,†], Keji Chen [†], Huixia Huang, Xinyang Zhong, Yaxian Wang, Jiayu Chen, Xuefeng He, Di Shi, Yupeng Zeng, Jiwei Li, Xiaoyan Zhou [*] and Ping Wei [*]

†These authors contributed equally to this work.

[*]Correspondence to:

Ping Wei (weiping@fudan.edu.cn) and Xiaoyan Zhou (xyzhou100@163.com); Tel: 021-64175590; Fax: 64172585; Cancer Institute, Fudan University Shanghai Cancer Center, 270 Dong'an Road, Shanghai, 200032, China.

Supplementary Figures

Figure S1. Flow chart of case selection and experimental design.

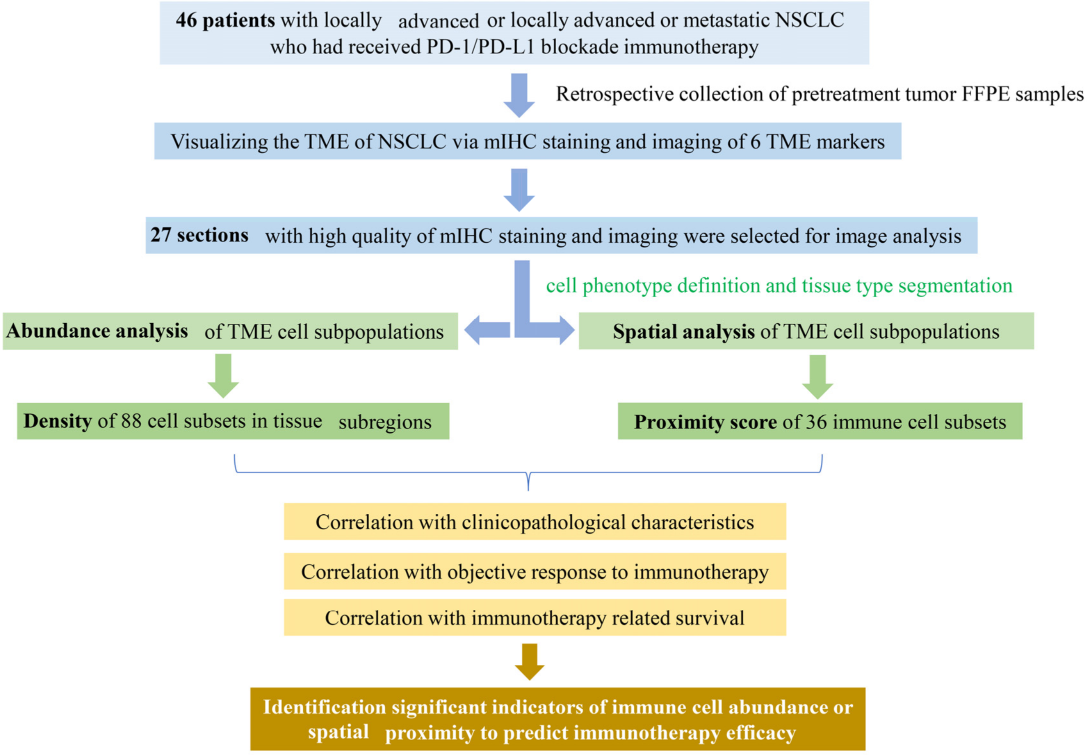

Figure S2. Overview of the image analysis method.

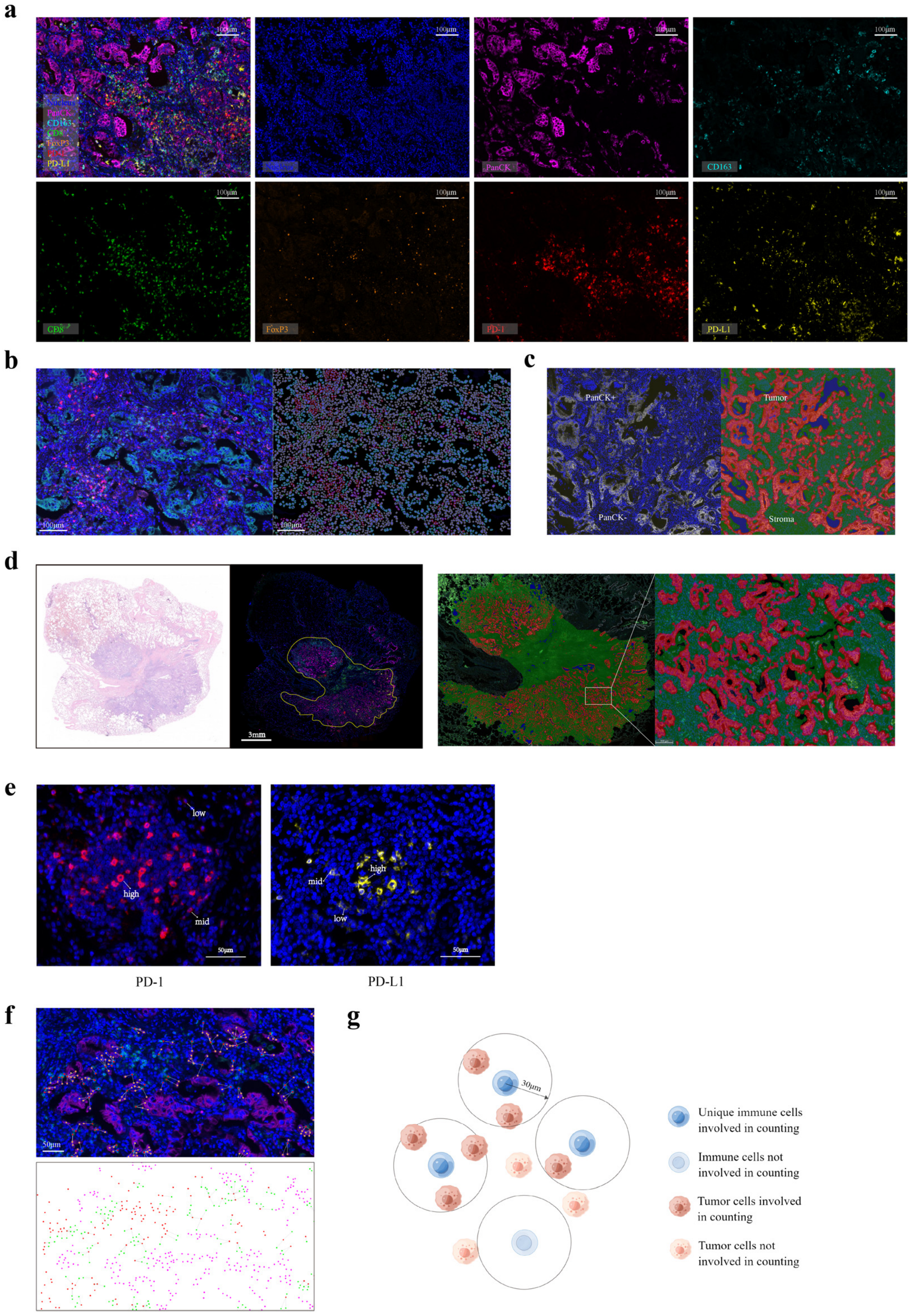

(a) Representative mIHC images of the TIME of advanced NSCLC; **(b)** Diagram of cell phenotype definition and segmentation; **(c)** Schematic diagram of tissue subregion definition based on PanCK signal; **(d)** An example of an automatic image analysis result; **(e)** Examples of differential expression and stratification of PD-1 and PD-L1 in cells; **(f)** Schematic of immune cell proximity scoring in mIHC images via HALO spatial analysis (real/simulated); **(g)** Schematic diagram of the calculation and definition of the proximity score.

Figure S3. Violin plot of tumoral density, stromal density and overall density of the immunocyte subpopulations.

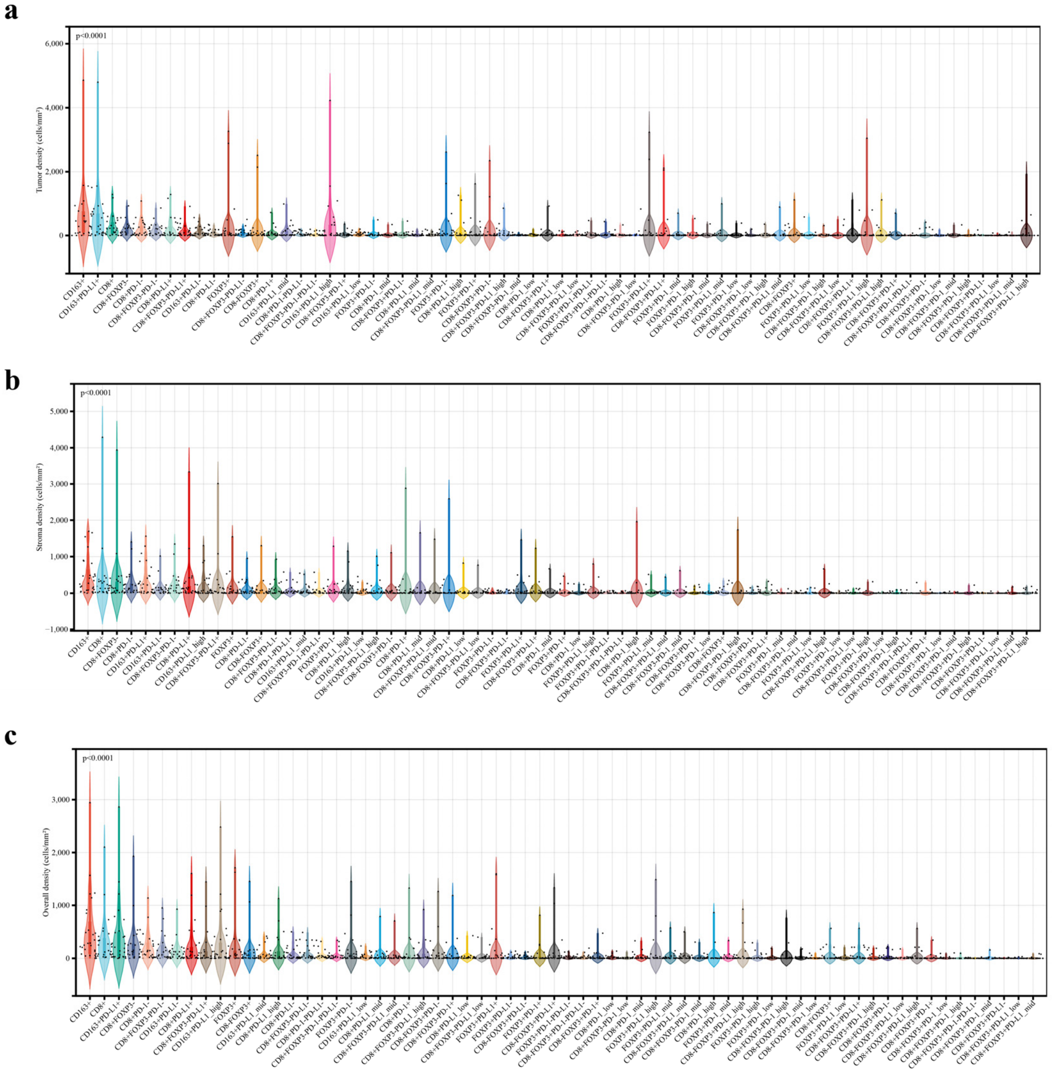

(a) Tumoral density; **(b)** Stromal density; **(c)** Overall density. The immunocyte subpopulations are in order of median density, and P-values for density difference analysis the cell subpopulations are also shown.

Figure S4. The results of the analysis of differences in the subregional abundance of each immunocyte subpopulation.

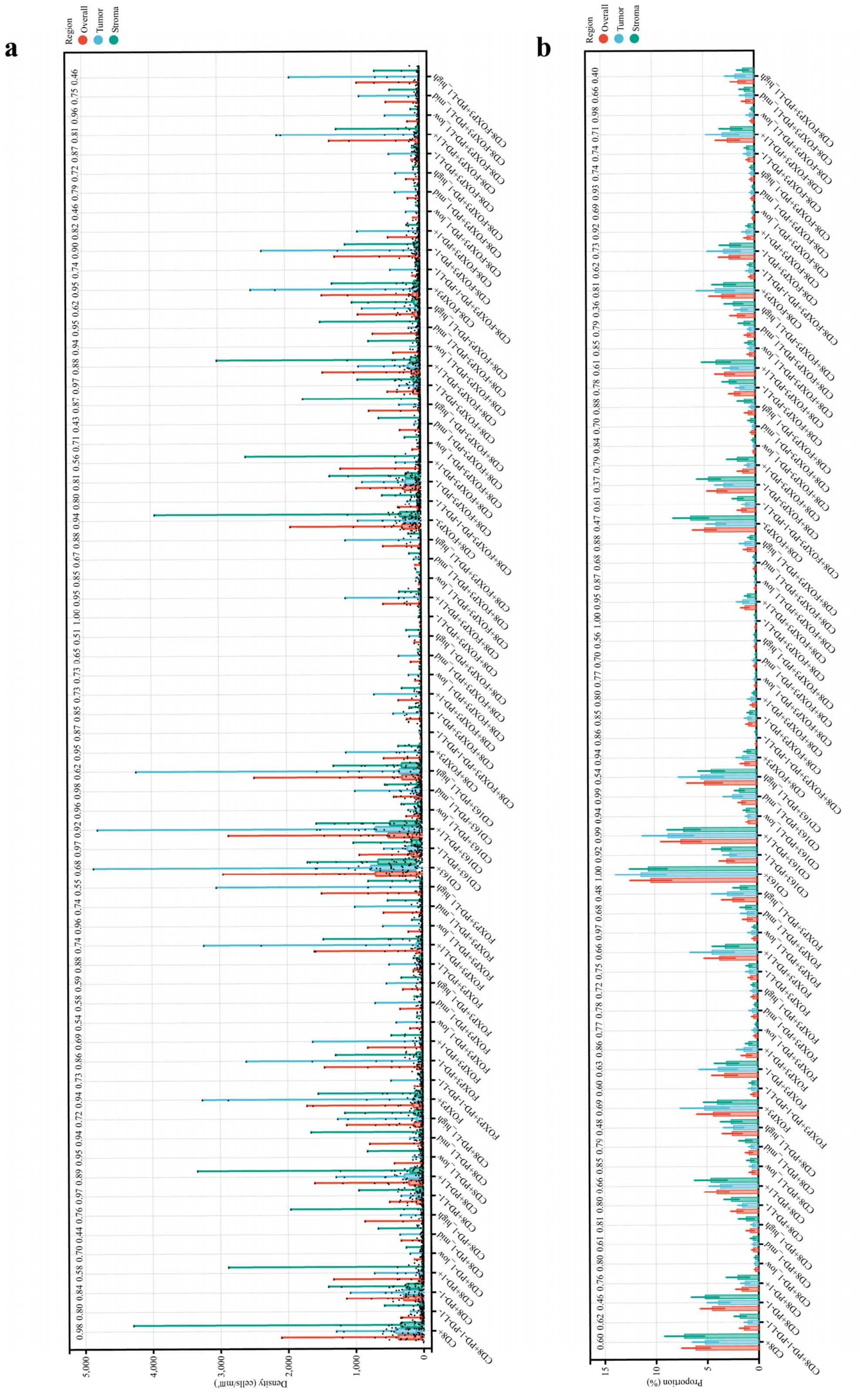

(a) Results of the analysis of differences in the density of each immunocyte subpopulation among different subregions; **(b)** Results of the analysis of differences in the proportion of each immunocyte subpopulation among different subregions.

Figure S5. Correlation between the densities of TIME cell subpopulations and clinicopathological factors.

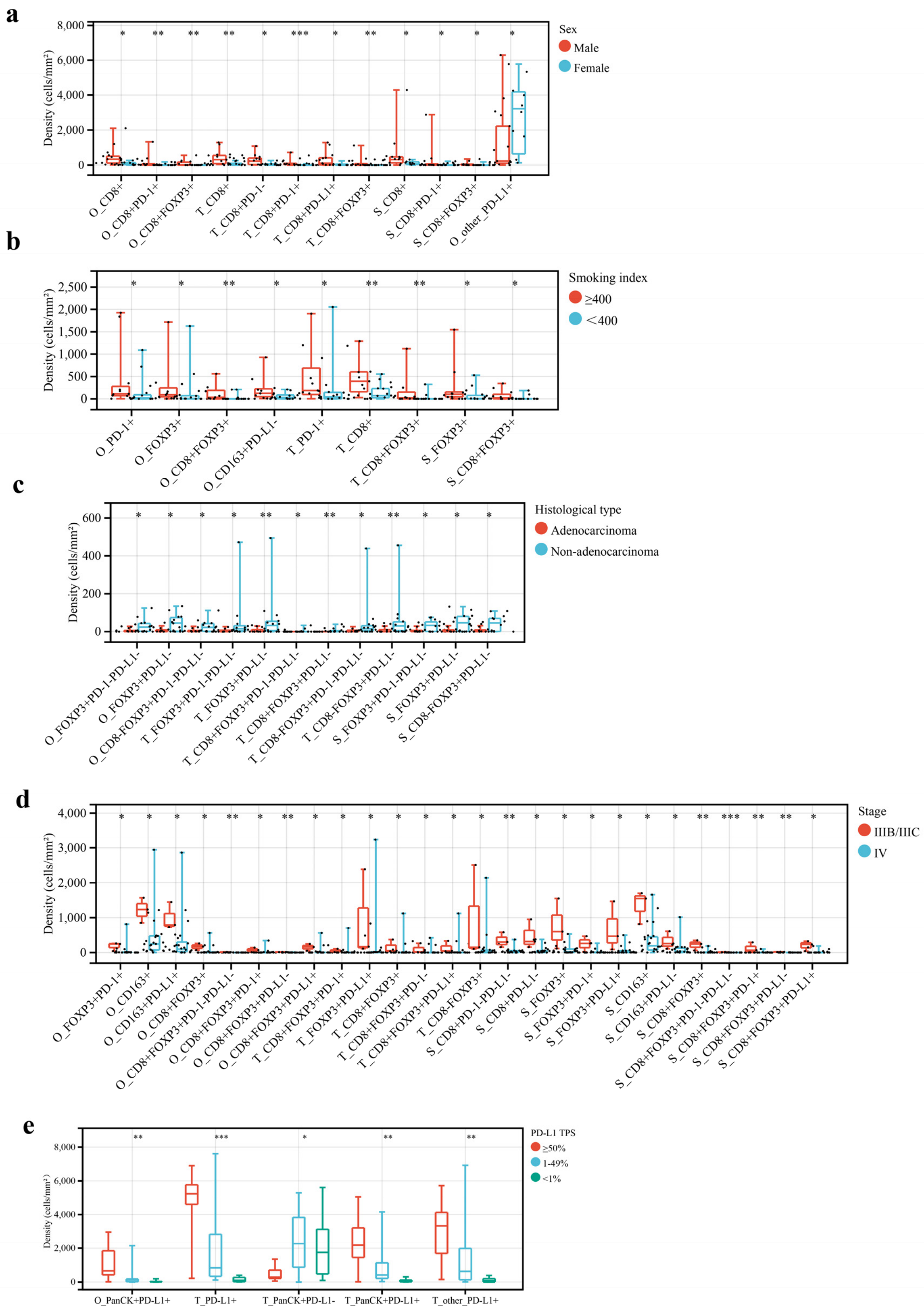

(a-d) Results of the analysis of intergroup differences in cell subpopulation densities significantly correlated with sex, smoking index, histological type and stage;**(e)** Results of comparing the consistency of PD-L1 expression in tumor cells detected by mIHC with clinical PD-L1 TPS (22C3).

Figure S6. Correlation between the proximity scores of immunocyte subpopulations and clinicopathological factors.

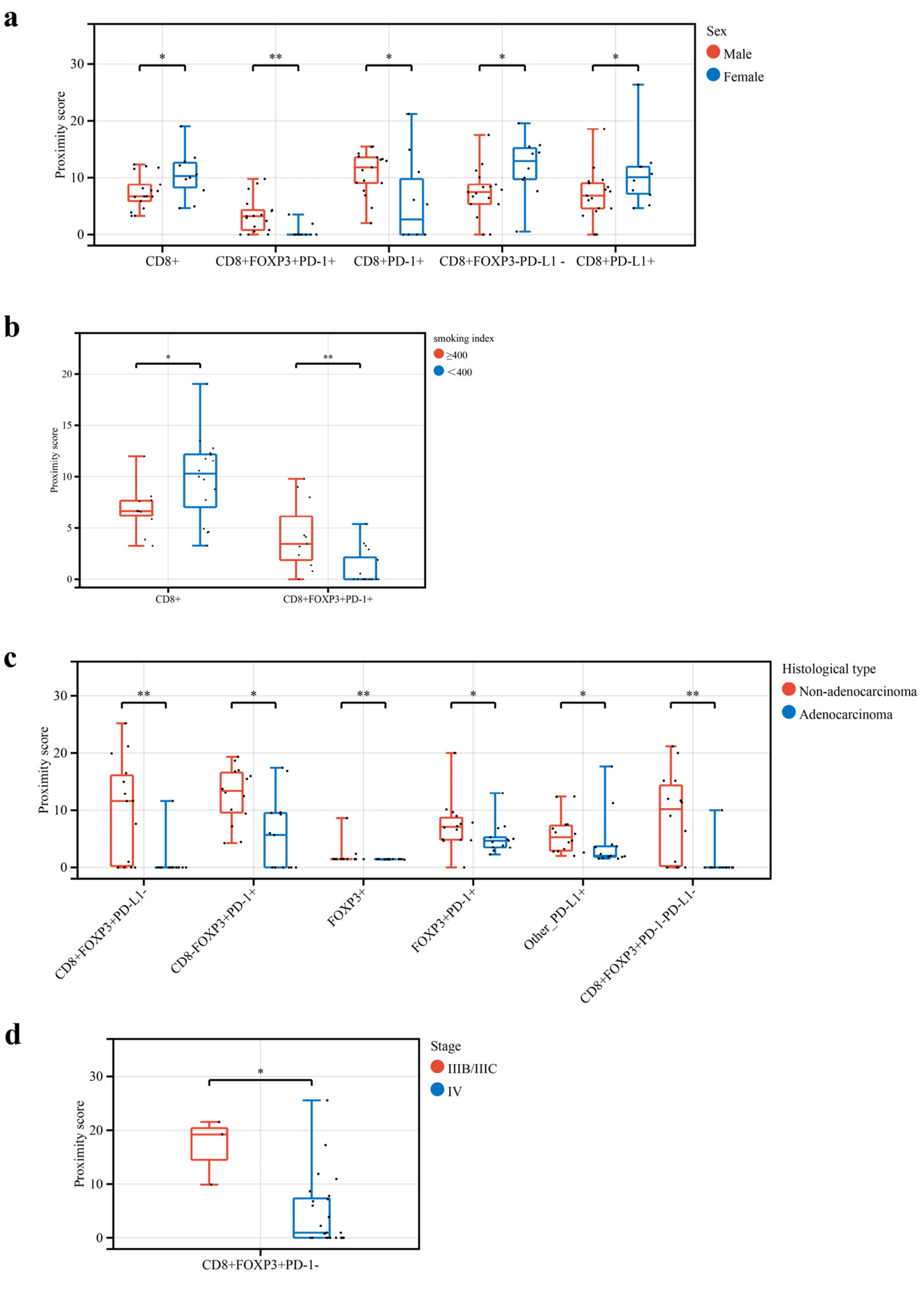

(a-d) Results of the analysis of intergroup differences in proximity scores significantly correlated with sex, smoking index, histological type and stage.

Figure S7. Results of the analysis of intergroup differences in cell subpopulation densities between responders and non-responders.

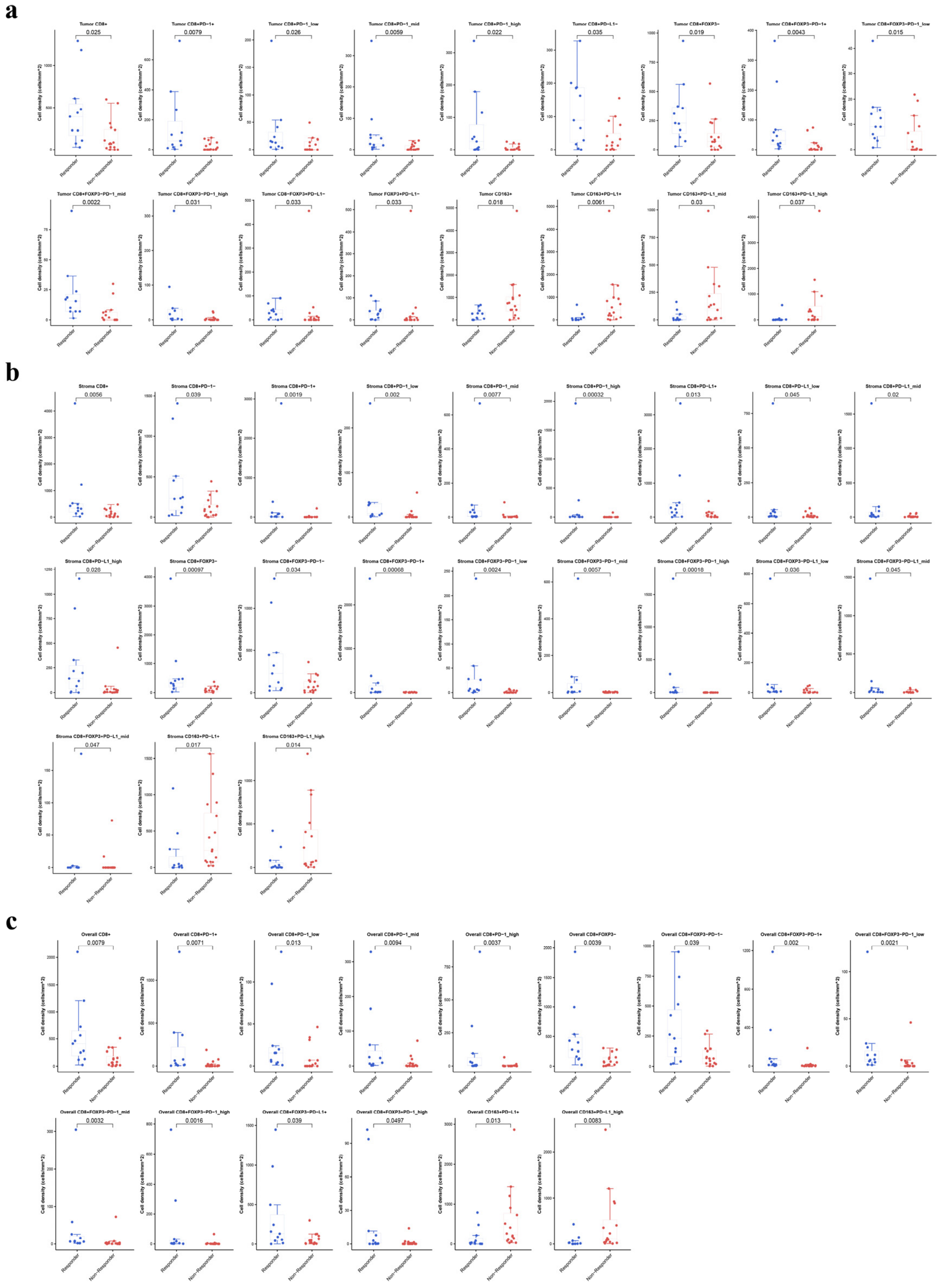

(a) Tumoral densities of 17 cell subpopulations significantly differed among patient subgroups; **(b)** Stromal densities of 21 cell subpopulations significantly differed among patient subgroups; **(c)** Overall densities of 15 cell subpopulations significantly differed among patient subgroups.

Figure S8. Significant results of the analysis of intergroup differences in immunocyte proximity scores between responders and non-responders.

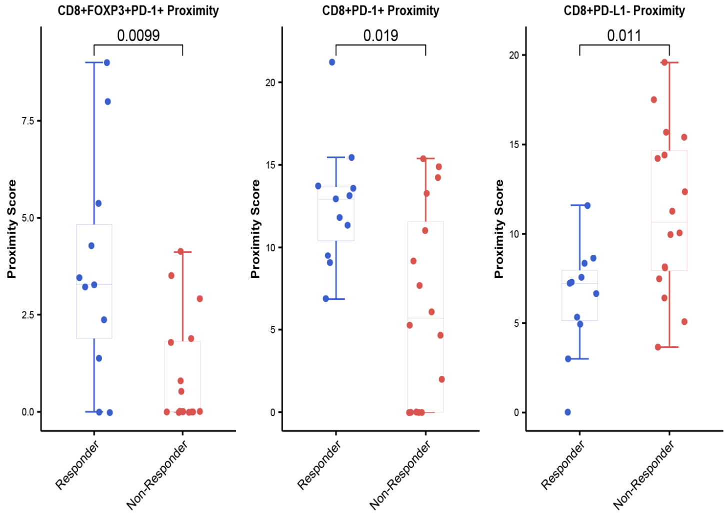

Figure S9. An overview of the distribution of TIME features in the population within the advanced NSCLC cohort.

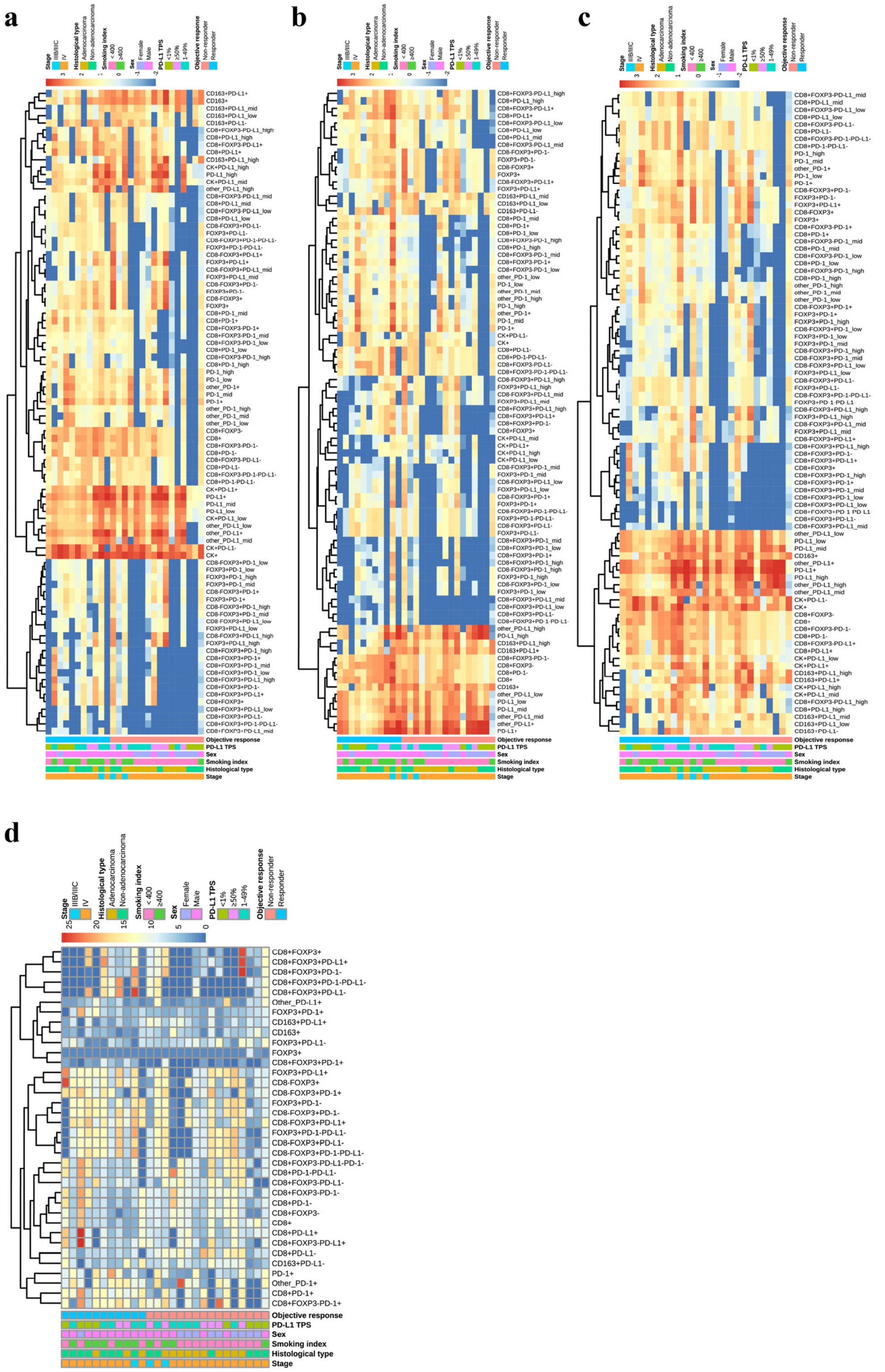

(a-c) Heatmap of the tumoral, stromal and overall densities of immunocyte subpopulations grouped by clinicopathologic subtypes and immunotherapy response; **(d)** Heatmap of the proximity scores of immunocyte subpopulations grouped by clinicopathologic subtypes and immunotherapy response.

Figure S10. Predictive value of densities of immunocyte subpopulations for immunotherapy-related survival.

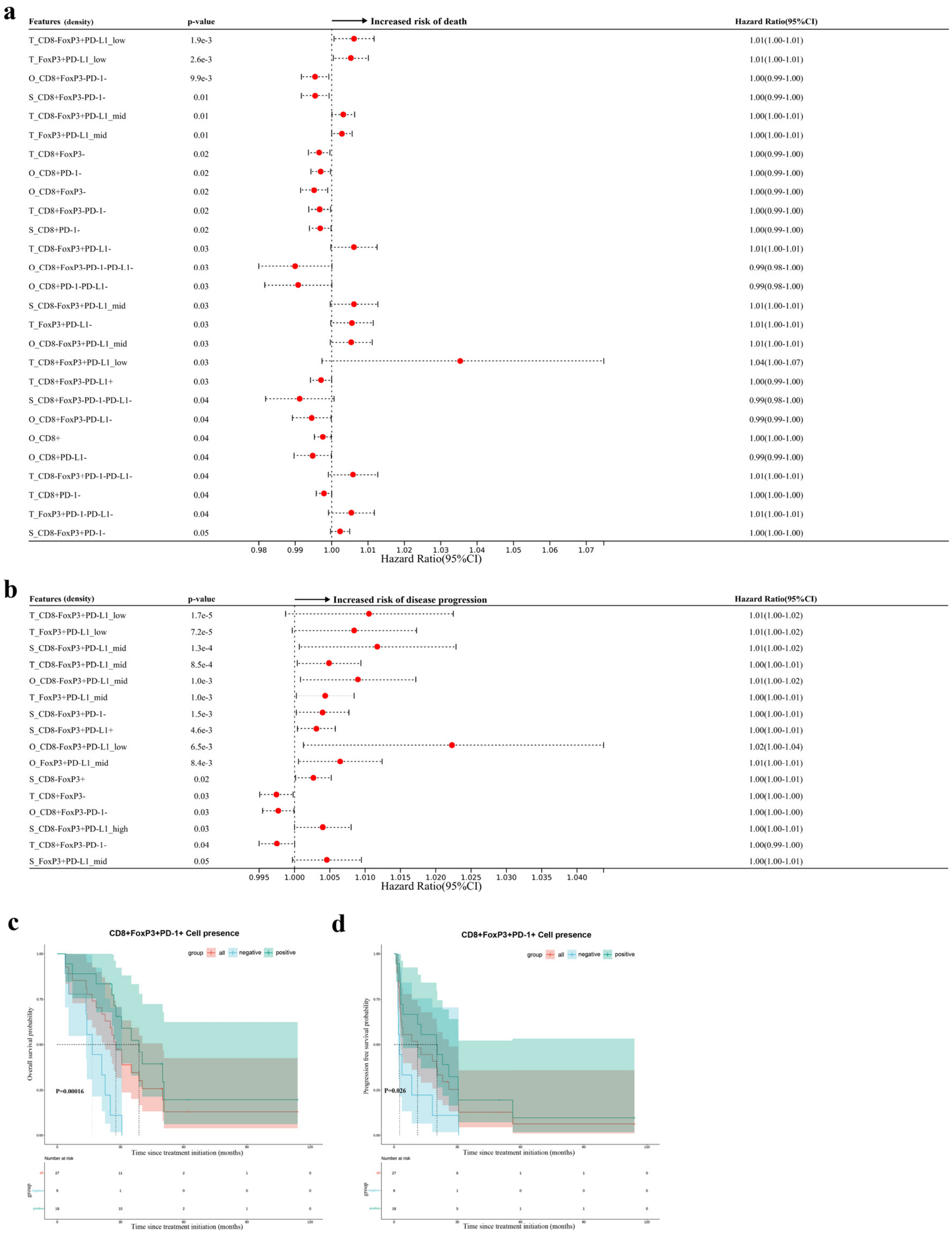

(a) Forest plot of the results of univariate Cox regression screening for immunocyte subregional densities significantly associated with OS; **(b)** Forest plot of the results of univariate Cox regression screening for immunocyte subregional densities significantly associated with PFS; **(c)** Survival analysis of $CD8^+FoxP3^+PD\text{-}1^+$ cell presence for OS; **(d)** Survival analysis of $CD8^+FoxP3^+PD\text{-}1^+$ cell presence for PFS.

Figure S11. Predictive value of proximity scores of immunocyte subpopulations for immunotherapy-related OS.

a

b

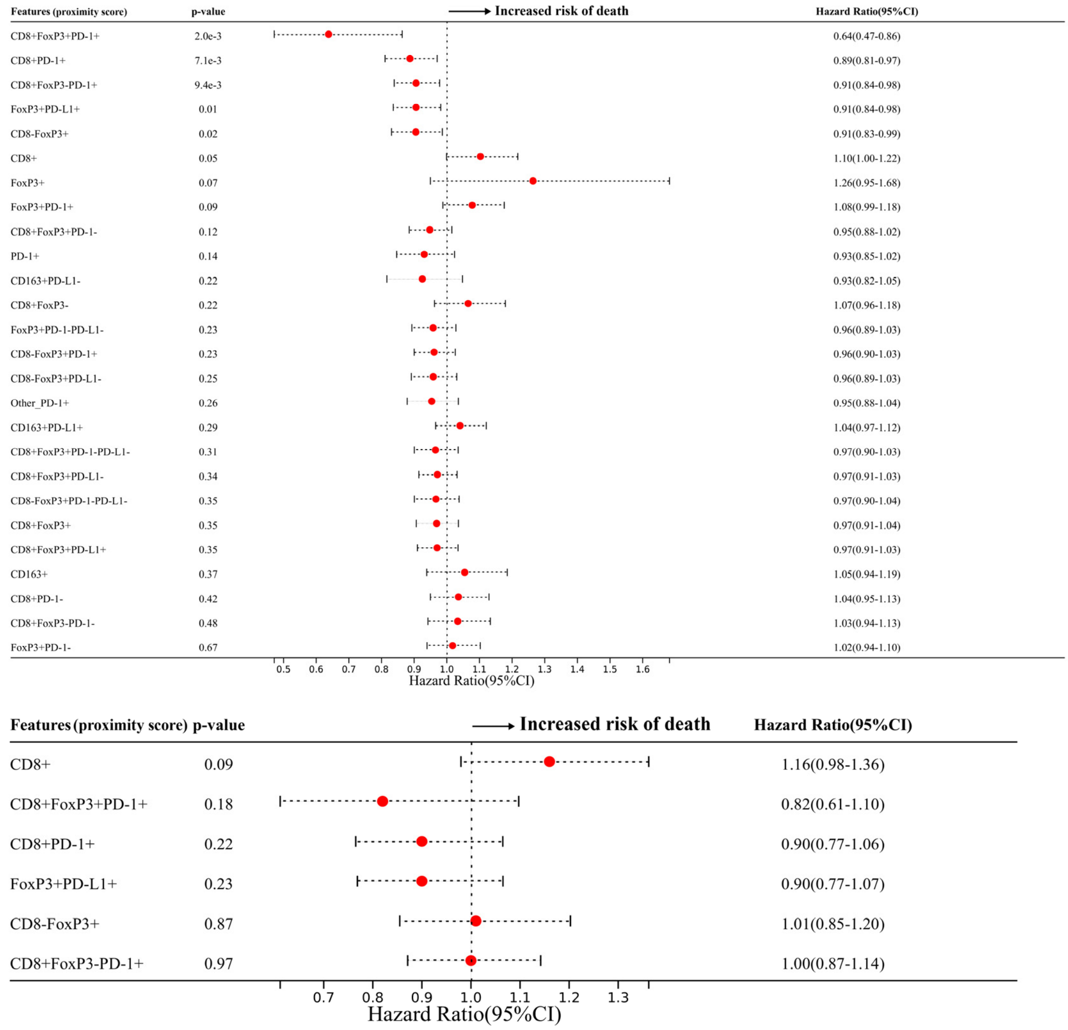

(a) Forest plot of the results from the univariate Cox regression analysis for immunocyte proximity scores associated with OS; **(b)** Forest plot of the results from the multivariate Cox regression analysis of immunocyte proximity scores significant in univariate analysis.

Figure S12. Forest plot of the results from the univariate Cox regression analysis of PFS including clinicopathological characteristics, immunotherapeutic regimens and CD8^{+}FoxP3^{+}PD-1^{+} cell proximity score (patients were grouped by the median proximity score).

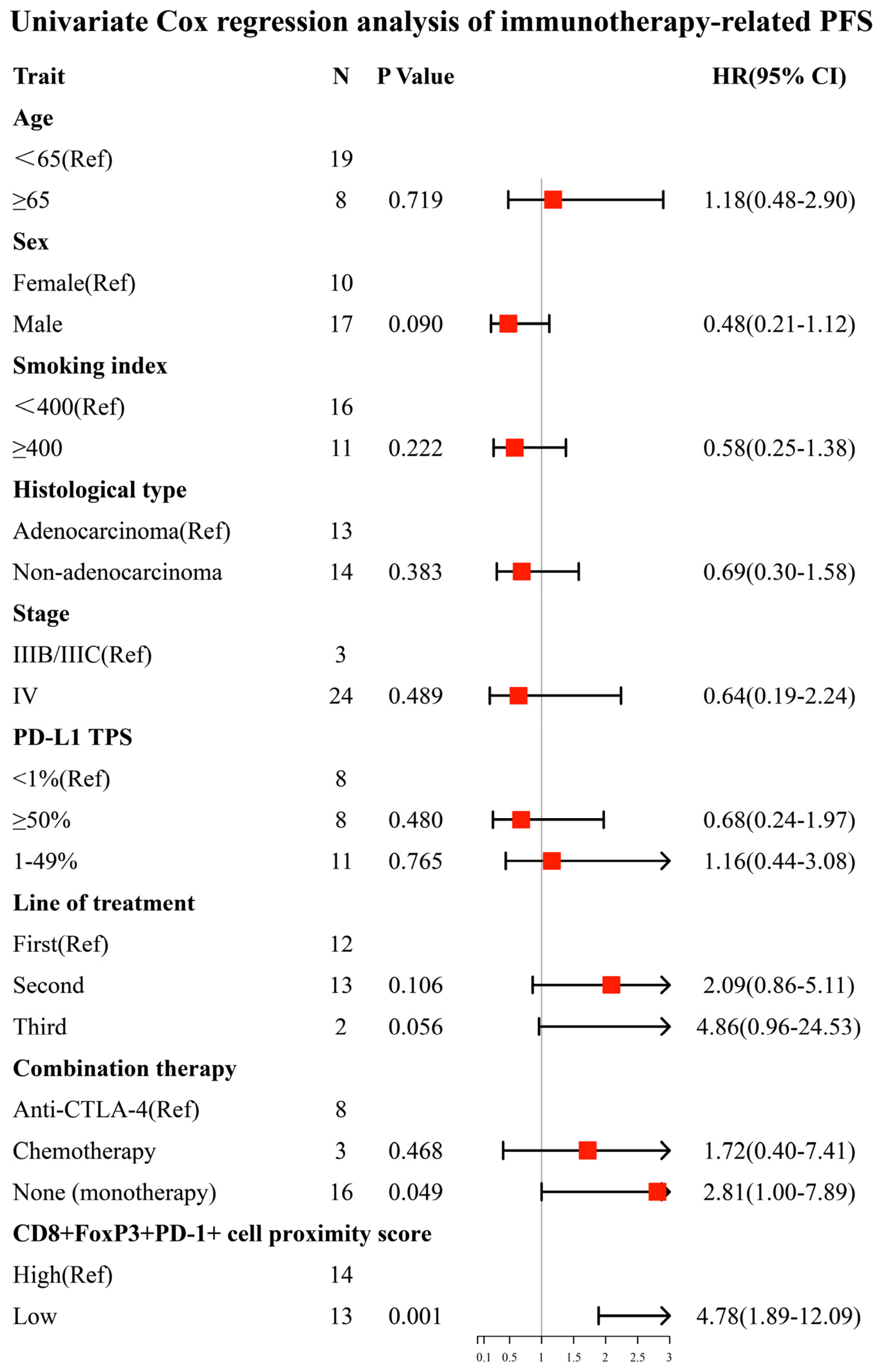

Supplementary Tables

Table S1. Experimental protocol for mIHC staining.

Dyeing sequence	Targets	Primary antibody dilution ratio	Fluorescent dye
1	PD-L1	1:100	Opal 570
2	CD8	1:200	Opal 520
3	PD-1	1:300	Opal 690
4	FoxP3	1:200	Opal 620
5	CD163	1:600	Opal 480
6	panCK	1:100	Opal 780
7	DNA (nucleus)	-	DAPI

Table S2. Clinicopathologic features and survival outcomes for individual patients.

Patient ID	Immunotherapeutic Response	Age	Sex	Line of Treatment	Treatment	OS (months)	OS Status	PFS (months)	PFS Status
P1	Non-Responder	73	Male	2nd	Nivolumab	3.97	Dead	0.93	Progressor
P2	Responder	60	Male	1st	Durvalumab & Tremelimumab	61.87	Alive	56.30	Progressor
P3	Non-Responder	28	Male	2nd	Nivolumab	26.87	Dead	11.20	Progressor
P4	Non-Responder	61	Male	2nd	Pembrolizumab	50.63	Dead	20.50	Progressor
P5	Non-Responder	62	Male	2nd	Nivolumab	25.20	Dead	3.73	Progressor
P6	Non-Responder	57	Female	3rd	Nivolumab	16.60	Dead	1.83	Progressor
P7	Non-Responder	67	Female	2nd	Nivolumab	5.43	Dead	1.90	Progressor
P8	Responder	65	Male	3rd	Atezoluzumab	18.47	Dead	4.23	Progressor
P9	Responder	58	Female	1st	Pembrolizumab & Chemotherapy	30.53	Dead	30.47	Progressor
P10	Responder	68	Male	1st	Pembrolizumab & Chemotherapy	25.97	Dead	25.97	Progressor
P11	Responder	51	Male	1st	Pembrolizumab & Chemotherapy	27.83	Dead	12.73	Progressor
P12	Non-Responder	44	Female	1st	Pembrolizumab & Chemotherapy	50.00	Dead	3.27	Progressor
P13	Responder	64	Male	1st	Pembrolizumab & Chemotherapy	49.80	Alive	49.80	Non-Progressor
P14	Responder	66	Male	2nd	Pembrolizumab & Chemotherapy	25.27	Alive	25.27	Non-Progressor
P15	Non-Responder	42	Male	2nd	Atezoluzumab	40.33	Dead	2.63	Progressor
P16	Responder	58	Male	2nd	Pembrolizumab & Chemotherapy	49.40	Alive	30.40	Progressor
P17	Responder	43	Male	1st	Nivolumab	114.07	Alive	114.07	Non-Progressor
P18	Non-Responder	48	Female	1st	Durvalumab & Tremelimumab	22.73	Dead	2.60	Progressor
P19	Responder	56	Male	1st	Pembrolizumab & Chemotherapy	28.73	Alive	20.13	Progressor
P20	Non-Responder	47	Female	1st	Nivolumab	35.13	Dead	23.00	Progressor
P21	Non-Responder	61	Male	2nd	Atezoluzumab	38.80	Dead	3.07	Progressor

P22	Non-Responder	57	Female	2nd	Atezoluzumab	13.67	Dead	1.53	Progressor
P23	Non-Responder	65	Female	2nd	Atezoluzumab	30.67	Dead	30.67	Progressor
P24	Non-Responder	50	Female	1st	Nivolumab	14.07	Dead	8.40	Progressor
P25	Non-Responder	53	Female	2nd	Nivolumab	3.73	Dead	2.10	Progressor
P26	Responder	65	Male	1st	Durvalumab & Tremelimumab	21.17	Dead	18.17	Progressor
P27	Non-Responder	68	Male	2nd	Atezoluzumab	7.20	Dead	2.80	Progressor

Table S3. Basic cell phenotypes for subpopulation analysis.

Single tagged	Double tagged	Triple tagged	Quadruple tagged	Five-plex tagged
panCK^{+}	panCK^{+}PD-L1^{-}	CD8^{+}PD-1^{-}PD-L1^{-}	CD8^{+}FoxP3^{+}PD-1^{-}PD-L1^{-}	other_PD-1^{+a}
CD163^{+}	panCK^{+}PD-L1^{+}	FoxP3^{+}PD-1^{-}PD-L1^{-}	CD8^{+}FoxP3^{-}PD-1^{-}PD-L1^{-}	other_PD-L1^{+b}
CD8^{+}	CD163^{+}PD-L1^{-}	CD8^{+}FoxP3^{+}PD-1^{-}	CD8^{-}FoxP3^{+}PD-1^{-}PD-L1^{-}	
FoxP3^{+}	CD163^{+}PD-L1^{+}	CD8^{+}FoxP3^{+}PD-1^{+}		
PD-1^{+}	CD8^{+}PD-1^{-}	CD8^{+}FoxP3^{+}PD-L1^{-}		
PD-L1^{+}	CD8^{+}PD-1^{+}	CD8^{+}FoxP3^{+}PD-L1^{+}		
	CD8^{+}PD-L1^{-}	CD8^{+}FoxP3^{-}PD-1^{-}		
	CD8^{+}PD-L1^{+}	CD8^{+}FoxP3^{-}PD-1^{+}		
	FoxP3^{+}PD-1^{-}	CD8^{+}FoxP3^{-}PD-L1^{-}		
	FoxP3^{+}PD-1^{+}	CD8^{+}FoxP3^{-}PD-L1^{+}		
	FoxP3^{+}PD-L1^{-}	CD8^{-}FoxP3^{+}PD-1^{-}		
	FoxP3^{+}PD-L1^{+}	CD8^{-}FoxP3^{+}PD-1^{+}		
	CD8^{+}FoxP3^{+}	CD8^{-}FoxP3^{+}PD-L1^{-}		
	CD8^{+}FoxP3^{-}	CD8^{-}FoxP3^{+}PD-L1^{+}		
	CD8^{-}FoxP3^{+}			

Table S4. Cell phenotype definition associated with differential PD-1/PD-L1 expression levels.

Single tagged	Double tagged	Triple tagged	Five-plex tagged
PD-1^{low}	$panCK^+$PD-$L1^{low}$	$CD8^+FoxP3^+$PD-1^{low}	other_PD-1^{low}
PD-1^{mid}	$panCK^+$PD-$L1^{mid}$	$CD8^+FoxP3^+$PD-1^{mid}	other_PD-1^{mid}
PD-1^{high}	$panCK^+$PD-$L1^{high}$	$CD8^+FoxP3^+$PD-1^{high}	other_PD-1^{high}
PD-$L1^{low}$	$CD163^+$PD-$L1^{low}$	$CD8^+FoxP3^+$PD-$L1^{low}$	other_PD-$L1^{low}$
PD-$L1^{mid}$	$CD163^+$PD-$L1^{mid}$	$CD8^+FoxP3^+$PD-$L1^{mid}$	other_PD-$L1^{mid}$
PD-$L1^{high}$	$CD163^+$PD-$L1^{high}$	$CD8^+FoxP3^+$PD-$L1^{high}$	other_PD-$L1^{high}$
	$CD8^+$PD-1^{low}	$CD8^+FoxP3^-$PD-1^{low}	
	$CD8^+$PD-1^{mid}	$CD8^+FoxP3^-$PD-1^{mid}	
	$CD8^+$PD-1^{high}	$CD8^+FoxP3^-$PD-1^{high}	
	$CD8^+$PD-$L1^{low}$	$CD8^+FoxP3^-$PD-$L1^{low}$	
	$CD8^+$PD-$L1^{mid}$	$CD8^+FoxP3^-$PD-$L1^{mid}$	
	$CD8^+$PD-$L1^{high}$	$CD8^+FoxP3^-$PD-$L1^{high}$	
	$FoxP3^+$PD-1^{low}	$CD8^-FoxP3^+$PD-1^{low}	
	$FoxP3^+$PD-1^{mid}	$CD8^-FoxP3^+$PD-1^{mid}	
	$FoxP3^+$PD-1^{high}	$CD8^-FoxP3^+$PD-1^{high}	
	$FoxP3^+$PD-$L1^{low}$	$CD8^-FoxP3^+$PD-$L1^{low}$	
	$FoxP3^+$PD-$L1^{mid}$	$CD8^-FoxP3^+$PD-$L1^{mid}$	
	$FoxP3^+$PD-$L1^{high}$	$CD8^-FoxP3^+$PD-$L1^{high}$	

Table S5. Results of difference analysis of tumor TIME cell subpopulation densities between responders and non-responders.

Features (density)	*P* value	Benjamini-Hochberg adjusted *p* value
Stroma $CD8^{+}FoxP3^{-}PD\text{-}1^{high}$	0.00018	0.0429
Stroma $CD8^{+}PD\text{-}1^{high}$	0.000325	0.0429
Stroma $CD8^{+}FoxP3^{-}PD\text{-}1^{+}$	0.000678	0.0564
Stroma $CD8^{+}FoxP3^{-}$	0.000971	0.0564
Overall $CD8^{+}FoxP3^{-}PD\text{-}1^{high}$	0.00157	0.0564
Stroma $CD8^{+}PD\text{-}1^{+}$	0.00193	0.0564
Stroma $CD8^{+}PD\text{-}1^{low}$	0.00199	0.0564
Overall $CD8^{+}FoxP3^{-}PD\text{-}1^{+}$	0.00201	0.0564
Overall $CD8^{+}FoxP3^{-}PD\text{-}1^{low}$	0.00211	0.0564
Tumor $CD8^{+}FoxP3^{-}PD\text{-}1^{mid}$	0.00221	0.0564
Stroma $CD8^{+}FoxP3^{-}PD\text{-}1^{low}$	0.00235	0.0564
Overall $CD8^{+}FoxP3^{-}PD\text{-}1^{mid}$	0.00323	0.07106
Overall $CD8^{+}PD\text{-}1^{high}$	0.0037	0.07392
Overall $CD8^{+}FoxP3^{-}$	0.00392	0.07392
Tumor $CD8^{+}FoxP3^{-}PD\text{-}1^{+}$	0.00434	0.076384
Stroma $CD8^{+}$	0.0056	0.085313684
Stroma $CD8^{+}FoxP3^{-}PD\text{-}1^{mid}$	0.00574	0.085313684
Tumor $CD8^{+}PD\text{-}1^{mid}$	0.0059	0.085313684
Tumor $CD163^{+}PD\text{-}L1^{+}$	0.00614	0.085313684
Overall $CD8^{+}PD\text{-}1^{+}$	0.00707	0.09108
Stroma $CD8^{+}PD\text{-}1^{mid}$	0.00775	0.09108
Overall $CD8^{+}$	0.00786	0.09108
Tumor $CD8^{+}PD\text{-}1^{+}$	0.00795	0.09108
Overall $CD163^{+}PD\text{-}L1^{high}$	0.00828	0.09108
Overall $CD8^{+}PD\text{-}1^{mid}$	0.00936	0.0988416
Overall $CD163^{+}PD\text{-}L1^{+}$	0.0127	0.124177778
Stroma $CD8^{+}PD\text{-}L1^{+}$	0.0127	0.124177778
Overall $CD8^{+}PD\text{-}1^{low}$	0.0133	0.1254
Stroma $CD163^{+}PD\text{-}L1^{high}$	0.0144	0.131089655
Tumor $CD8^{+}FoxP3^{-}PD\text{-}1^{low}$	0.0153	0.13464
Stroma $CD163^{+}PD\text{-}L1^{+}$	0.0167	0.142219355
Tumor $CD163^{+}$	0.0178	0.14685
Tumor $CD8^{+}FoxP3^{-}$	0.019	0.152
Stroma $CD8^{+}PD\text{-}L1^{mid}$	0.0197	0.152964706
Tumor $CD8^{+}PD\text{-}1^{high}$	0.0222	0.167451429
Tumor $CD8^{+}$	0.0247	0.181133333
Tumor $CD8^{+}PD\text{-}1^{low}$	0.0261	0.186227027
Stroma $CD8^{+}PD\text{-}L1^{high}$	0.0279	0.193831579
Tumor $CD163^{+}PD\text{-}L1^{mid}$	0.0297	0.201046154
Tumor $CD8^{+}FoxP3^{-}PD\text{-}1^{high}$	0.0314	0.204914286
Tumor $FoxP3^{+}PD\text{-}L1^{-}$	0.0326	0.204914286
Tumor $CD8^{-}FoxP3^{+}PD\text{-}L1^{-}$	0.0326	0.204914286

Stroma $CD8^{+}FoxP3^{-}PD\text{-}1^{-}$	0.0343	0.20944
Tumor $CD8^{+}PD\text{-}L1^{-}$	0.0354	0.20944
Stroma $CD8^{+}FoxP3^{-}PD\text{-}L1^{low}$	0.0357	0.20944
Tumor $CD163^{+}PD\text{-}L1^{high}$	0.0372	0.210122449
Overall $CD8^{+}FoxP3^{-}PD\text{-}1^{-}$	0.039	0.210122449
Overall $CD8^{+}FoxP3^{-}PD\text{-}L1^{+}$	0.039	0.210122449
Stroma $CD8^{+}PD\text{-}1^{-}$	0.039	0.210122449
Stroma $CD8^{+}FoxP3^{-}PD\text{-}L1^{mid}$	0.0445	0.234494118
Stroma $CD8^{+}PD\text{-}L1^{low}$	0.0453	0.234494118
Stroma $CD8^{+}FoxP3^{+}PD\text{-}L1^{mid}$	0.047	0.238615385
Overall $CD8^{+}FoxP3^{+}PD\text{-}1^{high}$	0.0497	0.24288
Overall $CD8^{+}PD\text{-}1^{-}$	0.0501	0.24288
Overall $CD8^{+}FoxP3^{-}PD\text{-}L1^{mid}$	0.0506	0.24288
Overall $CD8^{+}FoxP3^{+}PD\text{-}1^{+}$	0.0561	0.247986885
Overall $CD8^{+}PD\text{-}L1^{+}$	0.0565	0.247986885
Stroma $CD8^{+}FoxP3^{-}PD\text{-}L1^{+}$	0.0565	0.247986885
Overall $FoxP3^{+}PD\text{-}L1^{-}$	0.0567	0.247986885
Overall $CD8^{-}FoxP3^{+}PD\text{-}L1^{-}$	0.0567	0.247986885
Stroma $CD163^{+}PD\text{-}L1^{mid}$	0.0573	0.247986885
Tumor $CD8^{+}FoxP3^{+}PD\text{-}L1^{+}$	0.0632	0.26567619
Tumor $CD8^{+}FoxP3^{-}PD\text{-}L1^{+}$	0.0634	0.26567619
Overall $CD8^{+}FoxP3^{+}PD\text{-}L1^{mid}$	0.0694	0.283089231
Overall $CD8^{+}FoxP3^{+}PD\text{-}L1^{+}$	0.0697	0.283089231
Tumor $CD8^{+}PD\text{-}L1^{+}$	0.0708	0.2832
Overall $CD8^{+}FoxP3^{+}$	0.078	0.302823529
Overall $CD8^{+}FoxP3^{+}PD\text{-}L1^{high}$	0.078	0.302823529
Tumor $CD8^{+}FoxP3^{+}$	0.0795	0.304173913
Tumor $CD8^{+}PD\text{-}L1^{low}$	0.0859	0.316096
Overall $CD8^{+}PD\text{-}L1^{mid}$	0.0877	0.316096
Overall $CD8^{+}FoxP3^{-}PD\text{-}L1^{low}$	0.0885	0.316096
Tumor $CD8^{+}FoxP3^{+}PD\text{-}1^{+}$	0.0889	0.316096
Tumor $CD8^{+}FoxP3^{+}PD\text{-}L1^{high}$	0.0889	0.316096
Tumor $CD8^{-}FoxP3^{+}PD\text{-}1^{-}PD\text{-}L1^{-}$	0.0898	0.316096
Stroma $CD8^{+}FoxP3^{-}PD\text{-}L1^{-}$	0.0929	0.317436145
Tumor $CD163^{+}PD\text{-}L1^{low}$	0.0954	0.317436145
Tumor $CD8^{+}FoxP3^{-}PD\text{-}L1^{mid}$	0.0954	0.317436145
Stroma other_$PD\text{-}1^{low}$	0.0954	0.317436145
Overall $FoxP3^{+}PD\text{-}1^{-}PD\text{-}L1^{-}$	0.0973	0.317436145
Overall $CD163^{+}PD\text{-}L1^{mid}$	0.0981	0.317436145
Overall $CD8^{+}FoxP3^{+}PD\text{-}1^{low}$	0.0992	0.317436145
Tumor $FoxP3^{+}PD\text{-}1^{-}PD\text{-}L1^{-}$	0.0998	0.317436145
Tumor $CD8^{+}FoxP3^{+}PD\text{-}1^{high}$	0.105	0.327724138
Tumor $CD8^{+}PD\text{-}L1^{mid}$	0.106	0.327724138
Overall $CD8^{+}FoxP3^{+}PD\text{-}1^{-}$	0.108	0.327724138
Overall $CD8^{-}FoxP3^{+}PD\text{-}1^{-}PD\text{-}L1^{-}$	0.108	0.327724138
Tumor $CD8^{+}FoxP3^{-}PD\text{-}L1^{low}$	0.117	0.340645161

Tumor CD8$^+$FoxP3$^-$PD-L1$^-$	0.119	0.340645161
Overall CD8$^+$PD-L1^{low}	0.12	0.340645161
Overall other_PD-1^{high}	0.12	0.340645161
Tumor CD8$^+$FoxP3$^-$PD-1$^-$	0.12	0.340645161
Stroma other_PD-1$^+$	0.12	0.340645161
Overall CD8$^+$FoxP3$^+$PD-1^{mid}	0.13	0.345029703
Stroma PD-1^{low}	0.13	0.345029703
Tumor CD8$^+$FoxP3$^+$PD-L1^{mid}	0.131	0.345029703
Overall PD-1^{high}	0.132	0.345029703
Overall CD8$^+$FoxP3$^-$PD-L1^{high}	0.132	0.345029703
Overall other_PD-1$^+$	0.132	0.345029703
Tumor CD8$^+$PD-1$^-$	0.132	0.345029703
Stroma CD8$^+$FoxP3$^+$	0.132	0.345029703
Overall CD163$^+$	0.134	0.346823529
Tumor CD8$^+$FoxP3$^+$PD-1^{mid}	0.136	0.348582524
Stroma CD8$^+$FoxP3$^-$PD-L1^{high}	0.138	0.350307692
Stroma other_PD-1^{high}	0.142	0.35528972
Overall other_PD-1^{low}	0.143	0.35528972
Overall CD163$^+$PD-L1^{low}	0.144	0.35528972
Stroma CD163$^+$PD-L1^{low}	0.15	0.366666667
Overall CD8$^+$PD-L1^{high}	0.159	0.385100917
Stroma other_PD-L1^{high}	0.167	0.4008
Stroma PD-1^{high}	0.172	0.408626087
Stroma PD-1$^+$	0.174	0.408626087
Tumor CD8$^+$FoxP3$^+$PD-1$^-$	0.175	0.408626087
Stroma CD163$^+$	0.178	0.408626087
Stroma other_PD-L1^{mid}	0.178	0.408626087
Stroma PD-1^{mid}	0.19	0.432413793
Stroma CD8$^+$FoxP3$^+$PD-1$^-$	0.193	0.435487179
Tumor FoxP3$^+$	0.207	0.451636364
Tumor other_PD-1$^+$	0.207	0.451636364
Tumor other_PD-1^{high}	0.207	0.451636364
Stroma other_PD-1^{mid}	0.207	0.451636364
Overall PD-L1^{high}	0.212	0.45502439
Stroma PD-L1^{mid}	0.212	0.45502439
Stroma FoxP3$^+$PD-L1$^-$	0.223	0.466125
Stroma CD8$^-$FoxP3$^+$PD-L1$^-$	0.223	0.466125
Tumor CD8$^-$FoxP3$^+$	0.225	0.466125
Tumor CD8$^-$FoxP3$^+$PD-1$^-$	0.225	0.466125
Overall PD-1$^+$	0.226	0.466125
Stroma PD-L1^{high}	0.231	0.469107692
Stroma other_PD-L1$^+$	0.231	0.469107692
Stroma CD8$^+$FoxP3$^-$PD-1$^-$PD-L1$^-$	0.236	0.475603053
Overall other_PD-1^{mid}	0.246	0.490844444
Overall PD-L1^{mid}	0.251	0.490844444
Overall other_PD-L1^{mid}	0.251	0.490844444

Overall other_PD-L1^{high}	0.251	0.490844444
Stroma CD8^{+}FoxP3^{+}PD-L1^{+}	0.256	0.496941176
Overall FoxP3^{+}PD-1^{high}	0.263	0.503485714
Tumor CD8^{+}PD-1^{-}PD-L1^{-}	0.265	0.503485714
Tumor PD-1^{high}	0.266	0.503485714
Overall PD-1^{mid}	0.267	0.503485714
Tumor CD8^{+}FoxP3^{+}PD-L1^{low}	0.276	0.511384615
Tumor CD8^{+}FoxP3^{+}PD-L1^{-}	0.277	0.511384615
Stroma CD8^{+}FoxP3^{+}PD-L1^{-}	0.277	0.511384615
Tumor FoxP3^{+}PD-1^{-}	0.287	0.526166667
Tumor CD8^{+}FoxP3^{-}PD-L1^{high}	0.308	0.558530612
Overall CD8^{+}PD-L1^{-}	0.311	0.558530612
Overall CD8^{+}FoxP3^{-}PD-L1^{-}	0.311	0.558530612
Stroma CD8^{+}FoxP3^{+}PD-L1^{high}	0.325	0.57972973
Tumor other_PD-1^{low}	0.33	0.584697987
Overall PD-1^{low}	0.335	0.5896
Overall FoxP3^{+}PD-1^{+}	0.357	0.616
Tumor CD8^{+}PD-L1^{high}	0.357	0.616
Stroma FoxP3^{+}PD-1^{-}PD-L1^{-}	0.357	0.616
Overall PD-L1^{+}	0.368	0.626153846
Overall other_PD-L1^{+}	0.368	0.626153846
Stroma CD8^{+}FoxP3^{+}PD-L1^{low}	0.37	0.626153846
Stroma CD8^{+}FoxP3^{+}PD-1^{high}	0.377	0.633936306
Tumor PD-1^{+}	0.387	0.646632911
Stroma PD-L1^{+}	0.394	0.654188679
Stroma CD8^{-}FoxP3^{+}PD-1^{-}PD-L1^{-}	0.411	0.67815
Tumor FoxP3^{+}PD-1^{high}	0.418	0.685416149
Tumor FoxP3^{+}PD-L1^{+}	0.438	0.712481928
Tumor other_PD-L1^{high}	0.442	0.712481928
Stroma FoxP3^{+}	0.444	0.712481928
Overall FoxP3^{+}PD-L1^{low}	0.448	0.712481928
Stroma panCK^{+}PD-L1^{mid}	0.448	0.712481928
Stroma other_PD-L1^{low}	0.451	0.712958084
Stroma CD8^{+}PD-L1^{-}	0.458	0.719714286
Stroma CD8^{+}FoxP3^{+}PD-1^{+}	0.464	0.724828402
Tumor CD8^{-}FoxP3^{+}PD-L1^{+}	0.469	0.725614035
Overall CD8^{-}FoxP3^{+}PD-1^{+}	0.47	0.725614035
Overall FoxP3^{+}	0.474	0.727534884
Overall CD8^{-}FoxP3^{+}PD-L1^{low}	0.478	0.728275862
Stroma panCK^{+}PD-L1^{low}	0.48	0.728275862
Tumor other_PD-1^{mid}	0.484	0.730148571
Stroma CD8^{-}FoxP3^{+}PD-L1^{high}	0.497	0.744539326
Overall CD8^{-}FoxP3^{+}PD-1^{high}	0.5	0.744539326
Stroma CD8^{-}FoxP3^{+}PD-L1^{mid}	0.502	0.744539326
Tumor CD8^{+}FoxP3^{+}PD-1^{low}	0.524	0.770478261
Tumor FoxP3^{+}PD-L1^{mid}	0.53	0.770478261

Stroma FoxP3^{+}PD-L1^{mid}	0.534	0.770478261
Tumor PD-1^{mid}	0.536	0.770478261
Overall CD8^{-}FoxP3^{+}	0.537	0.770478261
Tumor PD-1^{low}	0.537	0.770478261
Tumor FoxP3^{+}PD-1^{+}	0.563	0.803416216
Overall CD163^{+}PD-L1^{-}	0.57	0.809032258
Tumor FoxP3^{+}PD-1^{low}	0.578	0.816
Tumor FoxP3^{+}PD-L1^{low}	0.592	0.821938144
Overall FoxP3^{+}PD-1^{low}	0.598	0.821938144
Tumor CD8^{-}FoxP3^{+}PD-1^{+}	0.598	0.821938144
Tumor CD8^{+}FoxP3^{-}PD-1^{-}PD-L1^{-}	0.603	0.821938144
Stroma CD8^{+}FoxP3^{+}PD-1^{-}PD-L1^{-}	0.603	0.821938144
Overall CD8^{+}FoxP3^{-}PD-1^{-}PD-L1^{-}	0.604	0.821938144
Overall CD8^{-}FoxP3^{+}PD-L1^{+}	0.604	0.821938144
Tumor panCK^{+}PD-L1^{low}	0.61	0.825673469
Tumor FoxP3^{+}PD-1^{mid}	0.613	0.825673469
Stroma FoxP3^{+}PD-1^{high}	0.624	0.831798995
Tumor CD8^{-}FoxP3^{+}PD-1^{high}	0.627	0.831798995
Stroma FoxP3^{+}PD-L1^{low}	0.627	0.831798995
Overall panCK^{+}PD-L1^{-}	0.645	0.842970297
Tumor PD-L1^{low}	0.645	0.842970297
Stroma PD-L1^{low}	0.645	0.842970297
Stroma FoxP3^{+}PD-1^{+}	0.669	0.860210526
Stroma FoxP3^{+}PD-L1^{high}	0.672	0.860210526
Overall CD8^{+}PD-1^{-}PD-L1^{-}	0.675	0.860210526
Tumor other_PD-L1^{+}	0.675	0.860210526
Overall panCK^{+}PD-L1^{+}	0.68	0.860210526
Overall other_PD-L1^{low}	0.68	0.860210526
Overall CD8^{+}FoxP3^{+}PD-L1^{low}	0.681	0.860210526
Stroma CD8^{-}FoxP3^{+}PD-L1^{low}	0.702	0.881239437
Tumor CD8^{-}FoxP3^{+}PD-L1^{mid}	0.706	0.881239437
Tumor PD-L1^{high}	0.711	0.881239437
Stroma CD8^{-}FoxP3^{+}	0.711	0.881239437
Tumor panCK^{+}PD-L1^{-}	0.716	0.88328972
Tumor CD8^{-}FoxP3^{+}PD-1^{low}	0.723	0.887776744
Tumor CD8^{-}FoxP3^{+}PD-L1^{low}	0.74	0.899511312
Overall CD8^{-}FoxP3^{+}PD-1^{low}	0.744	0.899511312
Stroma panCK^{+}	0.747	0.899511312
Stroma panCK^{+}PD-L1^{high}	0.75	0.899511312
Overall PD-L1^{low}	0.753	0.899511312
Overall panCK^{+}PD-L1^{low}	0.753	0.899511312
Overall FoxP3^{+}PD-1^{mid}	0.761	0.904972973
Overall CD8^{+}FoxP3^{+}PD-1^{-}PD-L1^{-}	0.769	0.906131004
Overall CD8^{+}FoxP3^{+}PD-L1^{-}	0.769	0.906131004
Overall panCK^{+}PD-L1^{high}	0.786	0.906131004
Overall FoxP3^{+}PD-1^{-}	0.786	0.906131004

Overall FoxP3$^+$PD-L1$^+$	0.786	0.906131004
Overall CD8$^-$FoxP3$^+$PD-1$^-$	0.786	0.906131004
Stroma CD8$^-$FoxP3$^+$PD-L1$^+$	0.786	0.906131004
Tumor panCK$^+$	0.79	0.906782609
Tumor CD8$^-$FoxP3$^+$PD-L1^{high}	0.807	0.922285714
Stroma FoxP3$^+$PD-1^{mid}	0.816	0.924566524
Stroma CD8$^-$FoxP3$^+$PD-1^{mid}	0.816	0.924566524
Stroma FoxP3$^+$PD-1$^-$	0.843	0.932081633
Stroma CD8$^+$FoxP3$^+$PD-1^{low}	0.846	0.932081633
Stroma CD8$^-$FoxP3$^+$PD-1^{low}	0.855	0.932081633
Stroma CD8$^-$FoxP3$^+$PD-1^{high}	0.855	0.932081633
Stroma CD8$^-$FoxP3$^+$PD-1$^+$	0.86	0.932081633
Overall FoxP3$^+$PD-L1^{mid}	0.863	0.932081633
Tumor panCK$^+$PD-L1^{high}	0.863	0.932081633
Tumor other_PD-L1^{mid}	0.863	0.932081633
Stroma FoxP3$^+$PD-L1$^+$	0.863	0.932081633
Stroma CD163$^+$PD-L1$^-$	0.863	0.932081633
Tumor PD-L1^{mid}	0.865	0.932081633
Tumor panCK$^+$PD-L1$^+$	0.865	0.932081633
Tumor CD8$^+$FoxP3$^+$PD-1$^-$PD-L1$^-$	0.873	0.936878049
Tumor CD163$^+$PD-L1$^-$	0.882	0.938903226
Stroma CD8$^+$PD-1$^-$PD-L1$^-$	0.882	0.938903226
Overall CD8$^-$FoxP3$^+$PD-1^{mid}	0.898	0.9504
Stroma panCK$^+$PD-L1$^+$	0.9	0.9504
Overall panCK$^+$	0.904	0.950820717
Stroma FoxP3$^+$PD-1^{low}	0.937	0.974211765
Tumor FoxP3$^+$PD-L1^{high}	0.939	0.974211765
Tumor CD8$^-$FoxP3$^+$PD-1^{mid}	0.939	0.974211765
Overall CD8$^-$FoxP3$^+$PD-L1^{mid}	0.941	0.974211765
Overall panCK$^+$PD-L1^{mid}	0.961	0.983348837
Tumor panCK$^+$PD-L1^{mid}	0.961	0.983348837
Stroma CD8$^-$FoxP3$^+$PD-1$^-$	0.961	0.983348837
Overall FoxP3$^+$PD-L1^{high}	0.98	0.987480916
Overall CD8$^-$FoxP3$^+$PD-L1^{high}	0.98	0.987480916
Tumor other_PD-L1^{low}	0.98	0.987480916
Stroma panCK$^+$PD-L1$^-$	0.98	0.987480916
Tumor PD-L1$^+$	1	1
Stroma CD8$^+$FoxP3$^+$PD-1^{mid}	1	1

Table S6. Results of difference analysis of proximity scores of immunocyte subpopulations between responders and non-responders.

Features (proximity score)	*P* value	Benjamini-Hochberg adjusted *p* value
$CD8^{+}FoxP3^{+}PD\text{-}1^{+}$	0.00988	0.1962
$CD8^{+}PD\text{-}L1^{-}$	0.0109	0.1962
$CD8^{+}PD\text{-}1^{+}$	0.0187	0.2244
$CD8^{-}FoxP3^{+}PD\text{-}L1^{+}$	0.0841	0.7569
$CD8^{+}$	0.11	0.792
$FoxP3^{+}PD\text{-}1^{+}$	0.195	0.799578947
$CD8^{+}FoxP3^{-}$	0.231	0.799578947
$FoxP3^{+}PD\text{-}L1$ -	0.231	0.799578947
$CD8^{+}FoxP3^{-}PD\text{-}1^{+}$	0.245	0.799578947
$PD\text{-}1^{+}$	0.246	0.799578947
$CD8^{+}FoxP3^{+}PD\text{-}L1^{-}$	0.332	0.799578947
$CD8^{-}FoxP3^{+}PD\text{-}1^{+}$	0.333	0.799578947
$FoxP3^{+}PD\text{-}L1^{+}$	0.335	0.799578947
$CD8^{-}FoxP3^{+}PD\text{-}1^{-}$	0.336	0.799578947
$FoxP3^{+}PD\text{-}1^{-}$	0.348	0.799578947
$CD8^{-}FoxP3^{+}$	0.368	0.799578947
Other_PD-1 $^{+}$	0.388	0.799578947
$CD163^{+}PD\text{-}L1^{+}$	0.415	0.799578947
$FoxP3^{+}$	0.422	0.799578947
$CD8^{+}FoxP3^{-}PD\text{-}1^{-}$	0.481	0.836571429
$CD8^{+}FoxP3^{+}PD\text{-}1^{-}PD\text{-}L1^{-}$	0.488	0.836571429
$CD163^{+}$	0.544	0.851478261
$CD8^{+}PD\text{-}1^{-}PD\text{-}L1^{-}$	0.544	0.851478261
$CD8^{-}FoxP3^{+}PD\text{-}1^{-}PD\text{-}L1^{-}$	0.621	0.89568
$CD8^{+}FoxP3^{-}PD\text{-}L1^{-}$	0.622	0.89568
$FoxP3^{+}PD\text{-}1^{-}PD\text{-}L1^{-}$	0.656	0.906206897
$CD8^{-}FoxP3^{+}PD\text{-}L1^{-}$	0.692	0.906206897
Other_PD-L1^{+}	0.716	0.906206897
$CD8^{+}FoxP3^{-}PD\text{-}L1^{+}$	0.73	0.906206897
$CD8^{+}FoxP3^{-}PD\text{-}L1^{-}PD\text{-}1^{-}$	0.79	0.917419355
$CD8^{+}PD\text{-}1^{-}$	0.79	0.917419355
$CD8^{+}FoxP3^{+}PD\text{-}1^{-}$	0.82	0.9225
$CD8^{+}FoxP3^{+}$	0.899	0.947314286
$CD8^{+}FoxP3^{+}PD\text{-}L1^{+}$	0.9	0.947314286
$CD8^{+}PD\text{-}L1^{+}$	0.921	0.947314286
$CD163^{+}PD\text{-}L1^{-}$	1	1

Table S7. Univariate Cox regression analysis to assess the correlation between the cell subpopulation densities and immunotherapy-related OS.

**Features (density)**	**HR[a] (95%CI[b])**	***P* value[c]**
Tumor CD8$^-$FoxP3$^+$PD-L1^{low}	1.0062 (1.0006-1.0117)	0.0019
Tumor FoxP3$^+$PD-L1^{low}	1.0053 (1.0005-1.0101)	0.0026
Overall CD8$^+$FoxP3$^-$PD-1$^-$	0.9954 (0.9917-0.9992)	0.0099
Stroma CD8$^+$FoxP3$^-$PD-1$^-$	0.9955 (0.9917-0.9993)	0.0123
Tumor CD8$^-$FoxP3$^+$PD-L1^{mid}	1.0032 (1.0001$^-$1.0063)	0.0124
Tumor FoxP3$^+$PD-L1^{mid}	1.0028 (1$^-$1.0056)	0.0144
Tumor CD8$^+$FoxP3$^-$	0.9966 (0.9936-0.9995)	0.0152
Overall CD8$^+$PD-1$^-$	0.997 (0.9943$^-$0.9997)	0.0191
Overall CD8$^+$FoxP3$^-$	0.9952 (0.9915-0.9989)	0.0197
Tumor CD8$^+$FoxP3$^-$PD-1$^-$	0.9967 (0.9937-0.9997)	0.0215
Stroma CD8$^+$PD-1$^-$	0.9969 (0.9939-0.9998)	0.022
Tumor CD8$^-$FoxP3$^+$PD-L1$^-$	1.0061 (0.9998$^-$1.0125)	0.0257
Overall CD8$^+$FoxP3$^-$PD-1$^-$PD-L1$^-$	0.99 (0.98$^-$1.0001)	0.0261
Overall CD8$^+$PD-1$^-$PD-L1$^-$	0.9908 (0.9816-1.0001)	0.0273
Stroma CD8$^-$FoxP3$^+$PD-L1^{mid}	1.0061 (0.9996-1.0127)	0.028
Tumor FoxP3$^+$PD-L1$^-$	1.0056 (0.9998$^-$1.0114)	0.0287
Overall CD8$^-$FoxP3$^+$PD-L1^{mid}	1.0054 (0.9996-1.0112)	0.0319
Tumor CD8$^+$FoxP3$^+$PD-L1^{low}	1.0354 (0.9973$^-$1.0748)	0.0327
Tumor CD8$^+$FoxP3$^-$PD-L1$^+$	0.9971 (0.9942-1)	0.035
Stroma CD8$^+$FoxP3$^-$PD-1$^-$PD-L1$^-$	0.9912 (0.9818$^-$1.0007)	0.0354
Overall CD8$^+$FoxP3$^-$PD-L1$^-$	0.9945 (0.9892-0.9999)	0.0356
Overall CD8$^+$	0.9976 (0.9953$^-$0.9999)	0.0373
Overall CD8$^+$PD-L1$^-$	0.9948 (0.9897-0.9999)	0.0379
Tumor CD8$^-$FoxP3$^+$PD-1$^-$PD-L1$^-$	1.0059 (0.9991$^-$1.0127)	0.0417
Tumor CD8$^+$PD-1$^-$	0.9979 (0.9958$^-$1)	0.0431
Tumor FoxP3$^+$PD-1$^-$PD-L1$^-$	1.0054 (0.9991$^-$1.0118)	0.0445
Stroma CD8$^-$FoxP3$^+$PD-1$^-$	1.0023 (0.9997-1.005)	0.0481
Overall CD8$^-$FoxP3$^+$PD-L1^{low}	1.0142 (0.9976-1.0312)	0.0578
Tumor CD8$^+$FoxP3$^-$PD-L1^{mid}	0.987 (0.9727-1.0014)	0.0624
Tumor CD8$^+$FoxP3$^-$PD-L1^{high}	0.9967 (0.993$^-$1.0005)	0.0639
Overall CD8$^+$FoxP3$^-$PD-L1$^+$	0.9973 (0.9939-1.0007)	0.0659
Stroma CD8$^+$PD-L1^{high}	0.9976 (0.9947-1.0006)	0.0686
Tumor CD8$^+$FoxP3$^-$PD-1^{low}	0.9386 (0.8763$^-$1.0052)	0.0712
Overall CD163$^+$PD-L1$^-$	0.9953 (0.99-1.0006)	0.0736
Stroma CD8$^+$FoxP3$^-$PD-L1^{high}	0.9974 (0.994-1.0007)	0.0752
Stroma CD8$^+$FoxP3$^-$	0.9958 (0.9925-0.9992)	0.0761
Stroma CD8$^-$FoxP3$^+$PD-L1$^+$	1.0019 (0.9996-1.0042)	0.0764
Tumor CD8$^+$	0.9985 (0.9968$^-$1.0002)	0.0786
Stroma CD8$^+$FoxP3$^-$PD-L1$^-$	0.9958 (0.9911$^-$1.0006)	0.08
Stroma CD8$^+$	0.9967 (0.9939-0.9995)	0.0817

Stroma CD163^{+}PD-L1^{-}	0.9962 (0.9916-1.0009)	0.0846
Stroma CD8^{+}PD-L1^{+}	0.9974 (0.9943-1.0005)	0.0899
Tumor CD8^{+}FoxP3^{+}PD-L1^{-}	1.0533 (0.9888-1.1219)	0.0901
Overall FoxP3^{+}PD-L1^{mid}	1.004 (0.9988-1.0092)	0.0993
Stroma other_PD-L1^{mid}	1.0008 (0.9998-1.0017)	0.0994
Overall CD8^{+}FoxP3^{-}PD-L1^{high}	0.9973 (0.9936-1.0009)	0.1005
Tumor CD8^{+}FoxP3^{-}PD-1^{mid}	0.9606 (0.9163-1.0071)	0.1049
Tumor CD8^{+}PD-L1^{mid}	0.9908 (0.9794-1.0024)	0.1064
Tumor CD8^{+}FoxP3^{+}PD-1^{-}PD-L1^{-}	1.0625 (0.9806-1.1512)	0.1099
Overall CD8^{+}PD-L1^{+}	0.9986 (0.9968-1.0004)	0.1101
Stroma CD8^{+}FoxP3^{-}PD-L1^{+}	0.9975 (0.9942-1.0008)	0.1118
Tumor CD8^{+}PD-1^{-}PD-L1^{-}	0.9919 (0.9815-1.0024)	0.113
Stroma CD8^{-}FoxP3^{+}	1.0017 (0.9995-1.004)	0.1136
Stroma other_PD-L1^{+}	1.0002 (0.9999-1.0004)	0.1143
Stroma CD8^{-}FoxP3^{+}PD-L1^{high}	1.0029 (0.999-1.0069)	0.1291
Overall CD8^{+}FoxP3^{-}PD-1^{low}	0.9593 (0.9066-1.0151)	0.1386
Tumor CD8^{+}PD-L1^{+}	0.9989 (0.9973-1.0005)	0.1533
Overall other_PD-L1^{high}	1.0002 (0.9999-1.0005)	0.1572
Overall FoxP3^{+}PD-L1^{low}	1.0099 (0.9954-1.0247)	0.1582
Tumor CD8^{+}FoxP3^{+}PD-L1^{mid}	1.0191 (0.9909-1.0481)	0.163
Tumor CD163^{+}PD-L1^{mid}	1.0014 (0.9994-1.0035)	0.1708
Overall other_PD-L1^{+}	1.0002 (0.9999-1.0004)	0.1821
Stroma CD8^{+}PD-1^{-}PD-L1^{-}	0.996 (0.9899-1.0022)	0.1822
Overall CD8^{+}PD-L1^{high}	0.9987 (0.9967-1.0007)	0.183
Stroma CD8^{+}PD-L1^{-}	0.9974 (0.9934-1.0013)	0.1854
Tumor CD8^{+}PD-L1^{low}	0.9891 (0.9724-1.006)	0.1859
Stroma CD8^{+}PD-1^{low}	0.9732 (0.9379-1.0099)	0.1865
Stroma other_PD-L1^{high}	1.0002 (0.9999-1.0005)	0.1873
Stroma CD8^{+}FoxP3^{+}PD-1^{-}	0.9921 (0.9802-1.0041)	0.1888
Tumor CD163^{+}PD-L1^{low}	1.0056 (0.997-1.0142)	0.199
Tumor CD8^{+}PD-L1^{-}	0.9955 (0.9885-1.0025)	0.2027
Overall CK^{+}PD-L1^{high}	0.9993 (0.9981-1.0005)	0.228
Overall CD8^{+}FoxP3^{-}PD-1^{mid}	0.9836 (0.9554-1.0126)	0.2309
Stroma CD8^{+}FoxP3^{+}	0.9953 (0.9876-1.0031)	0.2322
Overall CD8^{+}FoxP3^{-}PD-L1^{mid}	0.9814 (0.9581-1.0054)	0.2401
Overall CK^{+}PD-L1^{+}	0.9997 (0.9991-1.0002)	0.2421
Stroma CD8^{+}FoxP3^{+}PD-L1^{+}	0.9954 (0.9876-1.0033)	0.2436
Stroma CD8^{+}FoxP3^{+}PD-L1^{high}	0.9946 (0.9852-1.0041)	0.2548
Tumor CD8^{+}FoxP3^{-}PD-1^{-}PD-L1^{-}	0.9947 (0.9853-1.0041)	0.2564
Overall CD8^{+}FoxP3^{-}PD-L1^{low}	0.986 (0.9604-1.0122)	0.2611
Overall other_PD-L1^{mid}	1.0005 (0.9996-1.0013)	0.2675
Stroma CD8^{+}PD-L1^{mid}	0.9774 (0.9563-0.9991)	0.2691
Overall CK^{+}PD-L1^{low}	0.9984 (0.9954-1.0013)	0.2703
Tumor CD8^{+}FoxP3^{+}PD-1^{mid}	1.0034 (0.9971-1.0098)	0.2703

Tumor CD8^{+}PD-L1^{high}	0.9991 (0.9974-1.0007)	0.2737
Stroma CD8^{-}FoxP3^{+}PD-1^{low}	1.0266 (0.9789-1.0765)	0.2747
Stroma CD8^{+}PD-L1^{low}	0.9788 (0.9518-1.0066)	0.2777
Overall CD8^{+}PD-L1^{mid}	0.9908 (0.9736-1.0083)	0.2841
Tumor CD8^{+}FoxP3^{+}PD-1^{+}	1.0016 (0.9985-1.0047)	0.2874
Overall CD8^{+}PD-L1^{low}	0.9914 (0.9738-1.0093)	0.2897
Stroma CD8^{+}FoxP3^{-}PD-1^{low}	0.9832 (0.9469-1.0208)	0.3005
Stroma CD8^{+}PD-1^{mid}	0.9904 (0.9696-1.0116)	0.3009
Overall CD8^{+}FoxP3^{-}PD-1^{+}	0.9973 (0.9916-1.0032)	0.308
Stroma CD8^{+}FoxP3^{-}PD-L1^{mid}	0.9828 (0.959-1.0071)	0.3087
Tumor CD8^{+}FoxP3^{+}PD-1^{low}	1.0056 (0.9944-1.017)	0.314
Tumor CD8^{+}FoxP3^{+}PD-1^{high}	1.0061 (0.9936-1.0187)	0.3234
Stroma CD8^{+}FoxP3^{-}PD-L1^{low}	0.9857 (0.9562-1.0163)	0.3257
Tumor CD8^{-}FoxP3^{+}PD-1^{-}	1.0005 (0.9994-1.0016)	0.3293
Stroma FoxP3^{+}PD-L1^{mid}	1.0027 (0.9971-1.0084)	0.3315
Tumor CD163^{+}PD-L1^{-}	0.9983 (0.995-1.0017)	0.332
Overall CD8^{+}FoxP3^{+}PD-1^{low}	1.0112 (0.9879-1.0351)	0.3366
Stroma CD8^{+}PD-1^{+}	0.9982 (0.993-1.0035)	0.3425
Overall CK^{+}PD-L1^{mid}	0.9992 (0.9976-1.0009)	0.3523
Stroma FoxP3^{+}PD-1^{-}	1.0012 (0.9986-1.0039)	0.3587
Stroma CD8^{+}FoxP3^{-}PD-1^{mid}	0.9943 (0.9783-1.0105)	0.3608
Overall CD8^{+}FoxP3^{+}PD-1^{mid}	1.0064 (0.9922-1.0209)	0.3647
Tumor CD8^{+}FoxP3^{-}PD-L1^{-}	0.997 (0.9903-1.0036)	0.3685
Stroma PD-1^{mid}	0.9986 (0.9956-1.0017)	0.3722
Tumor CD8^{+}FoxP3^{-}PD-L1^{low}	0.9915 (0.9726-1.0107)	0.3734
Tumor CD8^{+}FoxP3^{-}PD-1^{+}	0.9959 (0.9865-1.0053)	0.3791
Overall CK^{+}	0.9999 (0.9995-1.0002)	0.3814
Stroma CD8^{+}FoxP3^{-}PD-1^{+}	0.9989 (0.9956-1.0022)	0.3843
Stroma CD8^{+}PD-1^{high}	0.9985 (0.9939-1.003)	0.387
Overall CD8^{+}FoxP3^{-}PD-1^{high}	0.9974 (0.991-1.0039)	0.3939
Stroma PD-1^{+}	0.9996 (0.9987-1.0006)	0.3961
Stroma CD8^{+}FoxP3^{-}PD-1^{high}	0.9986 (0.9945-1.0027)	0.4074
Overall CD8^{-}FoxP3^{+}PD-1^{-}	1.0009 (0.9987-1.0031)	0.4156
Stroma CD8^{+}FoxP3^{+}PD-1^{high}	0.9904 (0.9646-1.017)	0.4166
Overall CD8^{+}PD-1^{low}	0.9914 (0.9703-1.0129)	0.422
Overall CD8^{+}PD-1^{+}	0.9987 (0.9955-1.002)	0.4231
Stroma PD-1^{high}	0.9993 (0.9974-1.0012)	0.4255
Stroma CD8^{+}FoxP3^{+}PD-L1^{-}	0.9685 (0.8939-1.0494)	0.4273
Overall CD8^{+}PD-1^{mid}	0.9956 (0.9844-1.0069)	0.4301
Overall CD8^{+}PD-1^{high}	0.9981 (0.993-1.0032)	0.4369
Overall PD-L1^{mid}	1.0003 (0.9996-1.0009)	0.4475
Stroma PD-1^{low}	0.9984 (0.9942-1.0026)	0.4478
Stroma CD163^{+}	0.9997 (0.9989-1.0005)	0.45
Stroma CD8^{+}FoxP3^{+}PD-1^{-}PD-L1^{-}	0.9637 (0.872-1.0651)	0.4616

Tumor other_PD-1^{low}	1.001 (0.9983-1.0038)	0.4659
Overall CD8^{+}FoxP3^{+}PD-1^{-}	0.9972 (0.9896-1.0048)	0.4666
Stroma FoxP3^{+}PD-1^{low}	1.0102 (0.9824-1.0387)	0.4738
Stroma CD163^{+}PD-L1^{low}	0.9979 (0.9922-1.0037)	0.4745
Stroma PD-L1^{+}	1.0001 (0.9999-1.0003)	0.4794
Stroma PD-L1^{mid}	1.0002 (0.9996-1.0009)	0.483
Stroma PD-L1^{high}	1.0001 (0.9998-1.0004)	0.5054
Overall PD-L1^{+}	1.0001 (0.9999-1.0003)	0.5075
Stroma CD8^{-}FoxP3^{+}PD-L1^{low}	1.0078 (0.9847-1.0316)	0.5076
Stroma CK^{+}PD-L1^{-}	1.0037 (0.9926-1.015)	0.5084
Stroma other_PD-1^{mid}	0.9982 (0.9928-1.0036)	0.5086
Overall PD-L1^{high}	1.0001 (0.9998-1.0004)	0.5113
Tumor CD8^{-}FoxP3^{+}	1.0002 (0.9995-1.001)	0.5113
Stroma CK^{+}PD-L1^{high}	0.982 (0.9296-1.0373)	0.5126
Stroma CD8^{+}FoxP3^{+}PD-1^{+}	0.9963 (0.9847-1.008)	0.5207
Tumor other_PD-1^{+}	1.0004 (0.9991-1.0018)	0.5215
Overall PD-1^{mid}	0.999 (0.9959-1.0021)	0.5259
Stroma CD8^{+}FoxP3^{+}PD-L1^{low}	0.9825 (0.9284-1.0397)	0.532
Overall PD-1^{high}	0.9992 (0.9968-1.0017)	0.5329
Overall CD8^{-}FoxP3^{+}PD-L1^{+}	1.0005 (0.999-1.002)	0.5376
Tumor other_PD-1^{mid}	1.0011 (0.9975-1.0047)	0.5392
Overall CD8^{-}FoxP3^{+}	1.0005 (0.999-1.0019)	0.5394
Overall PD-1^{+}	0.9997 (0.9986-1.0007)	0.5447
Tumor PD-1^{low}	1.0007 (0.9984-1.003)	0.558
Stroma FoxP3^{+}PD-L1^{+}	1.0006 (0.9984-1.0028)	0.5635
Stroma CD163^{+}PD-L1^{mid}	0.9992 (0.9962-1.0021)	0.5718
Tumor CK^{+}PD-L1^{-}	1.0001 (0.9998-1.0003)	0.5738
Overall CD8^{+}FoxP3^{+}PD-1^{+}	1.002 (0.9951-1.0089)	0.5744
Tumor FoxP3^{+}PD-1^{-}	1.0003 (0.9993-1.0012)	0.5817
Stroma CD8^{+}FoxP3^{+}PD-L1^{mid}	0.9945 (0.9745-1.015)	0.5854
Tumor CD8^{+}FoxP3^{+}	1.0006 (0.9985-1.0026)	0.5995
Overall CD163^{+}	0.9998 (0.9992-1.0005)	0.6
Stroma other_PD-L1^{low}	1.0004 (0.999-1.0017)	0.6071
Tumor CD8^{-}FoxP3^{+}PD-L1^{+}	1.0002 (0.9994-1.001)	0.6083
Stroma other_PD-1^{+}	0.9995 (0.9976-1.0014)	0.6173
Tumor CD8^{+}FoxP3^{+}PD-L1^{+}	1.0005 (0.9984-1.0026)	0.6308
Stroma FoxP3^{+}	1.0005 (0.9984-1.0026)	0.6321
Stroma other_PD-1^{low}	0.9987 (0.9934-1.004)	0.6367
Stroma FoxP3^{+}PD-1^{-}PD-L1^{-}	0.9949 (0.9737-1.0165)	0.6394
Overall CD8^{+}FoxP3^{+}PD-1^{-}PD-L1^{-}	0.9774 (0.8863-1.0778)	0.6443
Overall CD163^{+}PD-L1^{low}	0.9981 (0.99-1.0063)	0.6557
Overall PD-1^{low}	0.9991 (0.9949-1.0033)	0.6682
Stroma CK^{+}PD-L1^{+}	0.9948 (0.9711-1.019)	0.6693
Tumor PD-L1^{high}	0.9999 (0.9997-1.0002)	0.6712

Tumor CK^{+}PD-L1^{high}	0.9999 (0.9993-1.0004)	0.6739
Stroma FoxP3^{+}PD-1^{high}	0.9982 (0.9898-1.0067)	0.6751
Tumor PD-L1^{mid}	1.0001 (0.9996-1.0007)	0.6873
Stroma CD163^{+}PD-L1^{high}	1.0002 (0.9991-1.0014)	0.6882
Stroma CD8^{-}FoxP3^{+}PD-1^{+}	1.0016 (0.9935-1.0098)	0.7001
Stroma CD8^{-}FoxP3^{+}PD-1^{-}PD-L1^{-}	0.9956 (0.9731-1.0186)	0.7047
Stroma CD8^{+}FoxP3^{+}PD-1^{mid}	0.9922 (0.9512-1.0349)	0.7141
Stroma CK^{+}	1.0018 (0.9921-1.0115)	0.7195
Overall CD8^{+}FoxP3^{+}PD-L1^{-}	0.9857 (0.9086-1.0694)	0.7287
Tumor other_PD-1^{high}	1.0013 (0.9939-1.0088)	0.7304
Overall FoxP3^{+}PD-1^{-}PD-L1^{-}	0.9959 (0.9726-1.0198)	0.7347
Tumor CD8^{+}FoxP3^{+}PD-L1^{high}	1.0004 (0.9982-1.0026)	0.7378
Tumor CD163^{+}PD-L1^{+}	1.0001 (0.9997-1.0004)	0.7395
Overall CD8^{-}FoxP3^{+}PD-1^{-}PD-L1^{-}	0.9957 (0.9703-1.0218)	0.7452
Overall CD8^{-}FoxP3^{+}PD-1^{high}	1.0015 (0.9926-1.0104)	0.7453
Stroma FoxP3^{+}PD-L1^{high}	1.0006 (0.9972-1.0039)	0.7454
Tumor FoxP3^{+}	1.0001 (0.9996-1.0006)	0.7457
Tumor PD-1^{+}	1.0001 (0.9994-1.0009)	0.7466
Overall CD163^{+}PD-L1^{mid}	0.9994 (0.996-1.0029)	0.7507
Stroma other_PD-1^{high}	0.9991 (0.9933-1.0049)	0.753
Stroma FoxP3^{+}PD-L1^{-}	0.9978 (0.9842-1.0116)	0.7557
Tumor CD8^{+}FoxP3^{-}PD-1^{high}	0.9985 (0.9889-1.0083)	0.7651
Overall other_PD-1^{mid}	0.9992 (0.9936-1.0047)	0.7669
Tumor CK^{+}	1 (0.9998-1.0002)	0.7707
Stroma CD8^{-}FoxP3^{+}PD-1^{high}	1.0021 (0.9869-1.0176)	0.7849
Tumor PD-1^{mid}	1.0003 (0.9984-1.0021)	0.7851
Overall CD8^{-}FoxP3^{+}PD-1^{low}	1.0024 (0.9836-1.0215)	0.8059
Tumor CK^{+}PD-L1^{+}	1 (0.9997-1.0003)	0.8075
Overall CD8^{-}FoxP3^{+}PD-1^{+}	1.0005 (0.9966-1.0044)	0.8188
Overall CD8^{+}FoxP3^{+}PD-L1^{low}	0.9925 (0.9304-1.0588)	0.8196
Overall CD8^{+}FoxP3^{+}PD-L1^{mid}	0.9968 (0.9698-1.0246)	0.8209
Overall FoxP3^{+}PD-1^{-}	1.0002 (0.9984-1.0021)	0.8215
Stroma CD8^{-}FoxP3^{+}PD-L1^{-}	0.9983 (0.9833-1.0135)	0.827
Stroma CD8^{-}FoxP3^{+}PD-1^{mid}	1.0027 (0.9789-1.027)	0.8272
Tumor FoxP3^{+}PD-L1^{+}	1.0001 (0.9995-1.0006)	0.8295
Tumor CK^{+}PD-L1^{low}	1.0001 (0.9988-1.0015)	0.8298
Overall CD8^{-}FoxP3^{+}PD-L1^{high}	1.0002 (0.9983-1.0021)	0.8308
Stroma CD8^{+}FoxP3^{+}PD-1^{low}	1.0065 (0.9467-1.0701)	0.8359
Tumor other_PD-L1^{low}	0.9999 (0.9987-1.001)	0.8372
Overall other_PD-1^{+}	0.9998 (0.9979-1.0017)	0.8393
Tumor CD163^{+}	1 (0.9997-1.0004)	0.8419
Overall CD8^{+}FoxP3^{+}	0.9995 (0.995-1.0041)	0.8434
Overall CK^{+}PD-L1^{-}	1 (0.9995-1.0004)	0.8436
Stroma CK^{+}PD-L1^{mid}	0.9948 (0.944-1.0484)	0.8452

Tumor CD8$^-$FoxP3$^+$PD-1^{high}	1.0005 (0.9953$^-$1.0057)	0.8529
Tumor FoxP3$^+$PD-L1^{high}	0.9999 (0.9992-1.0006)	0.8553
Overall CD8$^+$FoxP3$^+$PD-L1$^+$	0.9996 (0.9951$^-$1.0041)	0.8587
Stroma FoxP3$^+$PD-L1^{low}	1.0015 (0.9849-1.0184)	0.8592
Stroma FoxP3$^+$PD-1$^+$	0.9996 (0.995-1.0042)	0.8625
Overall other_PD-1^{low}	0.9996 (0.9946-1.0046)	0.8665
Overall FoxP3$^+$PD-L1^{high}	0.9999 (0.9985-1.0012)	0.8753
Tumor CK$^+$PD-L1^{mid}	0.9999 (0.9991$^-$1.0008)	0.8785
Tumor PD-L1$^+$	1 (0.9998$^-$1.0002)	0.8841
Tumor CD8$^+$PD-1^{low}	0.9988 (0.9826-1.0153)	0.8895
Overall CD8$^+$FoxP3$^+$PD-1^{high}	0.9985 (0.9775-1.02)	0.8902
Overall FoxP3$^+$	1.0001 (0.999-1.0012)	0.8951
Overall CD163$^+$PD-L1^{high}	1 (0.9994-1.0007)	0.8956
Overall FoxP3$^+$PD-L1$^+$	1.0001 (0.9989-1.0012)	0.8968
Overall other_PD-1^{high}	0.9996 (0.9932-1.006)	0.8979
Overall CD8$^+$FoxP3$^+$PD-L1^{high}	0.9997 (0.9952-1.0043)	0.9009
Overall CD8$^-$FoxP3$^+$PD-1^{mid}	1.0006 (0.9898$^-$1.0115)	0.9156
Tumor CD8$^-$FoxP3$^+$PD-1$^+$	1.0001 (0.998$^-$1.0022)	0.9175
Overall PD-L1^{low}	0.9999 (0.9989-1.001)	0.9183
Tumor other_PD-L1^{high}	1 (0.9995-1.0004)	0.9235
Overall FoxP3$^+$PD-1^{low}	1.0005 (0.9903$^-$1.0107)	0.9253
Tumor CD163$^+$PD-L1^{high}	1 (0.9996-1.0005)	0.9277
Overall other_PD-L1^{low}	1.0001 (0.9988$^-$1.0013)	0.9278
Tumor FoxP3$^+$PD-1^{high}	1.0002 (0.9965-1.0038)	0.9287
Tumor other_PD-L1$^+$	1 (0.9998$^-$1.0002)	0.932
Tumor PD-1^{high}	1.0001 (0.9977-1.0025)	0.9357
Overall CD8$^-$FoxP3$^+$PD-L1$^-$	1.0006 (0.9833$^-$1.0182)	0.9449
Tumor CD8$^+$FoxP3$^+$PD-1$^-$	0.9998 (0.9953$^-$1.0044)	0.9465
Tumor CD8$^+$PD-1^{mid}	1.0003 (0.9914-1.0093)	0.9487
Tumor CD8$^-$FoxP3$^+$PD-1^{low}	1.0003 (0.9906-1.0101)	0.9514
Tumor CD8$^+$PD-1^{high}	1.0002 (0.9932-1.0072)	0.9612
Overall FoxP3$^+$PD-1^{high}	0.9998 (0.9933$^-$1.0064)	0.9634
Tumor CD8$^-$FoxP3$^+$PD-1^{mid}	1.0001 (0.9947-1.0056)	0.9637
Stroma PD-L1^{low}	1 (0.9989-1.001)	0.9645
Tumor PD-L1^{low}	1 (0.999-1.0011)	0.9649
Overall FoxP3$^+$PD-L1$^-$	1.0003 (0.9849-1.0161)	0.965
Stroma CD163$^+$PD-L1$^+$	1 (0.9991$^-$1.0008)	0.9735
Tumor FoxP3$^+$PD-1$^+$	1 (0.9988$^-$1.0012)	0.9754
Tumor CD8$^-$FoxP3$^+$PD-L1^{high}	1 (0.999-1.001)	0.9782
Tumor other_PD-L1^{mid}	1 (0.9994-1.0007)	0.9796
Stroma FoxP3$^+$PD-1^{mid}	0.9998 (0.9847-1.0153)	0.9838
Tumor CD8$^+$PD-1$^+$	1 (0.9963$^-$1.0038)	0.9861
Overall CD163$^+$PD-L1$^+$	1 (0.9994-1.0006)	0.9867
Overall FoxP3$^+$PD-1^{mid}	0.9999 (0.9939-1.006)	0.9869

Tumor FoxP3$^+$PD-1^{low}	1 (0.9949-1.0051)	0.9938
Stroma CK$^+$PD-L1^{low}	1.0001 (0.9422-1.0616)	0.9962
Overall FoxP3$^+$PD-1$^+$	1 (0.9976-1.0024)	0.9981
Tumor FoxP3$^+$PD-1^{mid}	1 (0.9971$^-$1.0029)	0.9991

a.HR = Hazard Ratio, b.CI = Confidence Interval, c. The *p*-values were obtained from the log-rank test.

Table S8. Univariate Cox regression analysis to assess the correlation between the cell subpopulation densities and immunotherapy-related PFS.

**Features (density)**	**HR[a] (95%CI[b])**	***P* value[c]**
Tumor CD8$^-$FoxP3$^+$PD-L1^{low}	1.0106 (0.9987-1.0225)	0.000017
Tumor FoxP3$^+$PD-L1^{low}	1.0084 (0.9997-1.0173)	0.0000721
Stroma CD8$^-$FoxP3$^+$PD-L1^{mid}	1.0117 (1.0007-1.0229)	0.0001
Tumor CD8$^-$FoxP3$^+$PD-L1^{mid}	1.0049 (1.0004-1.0094)	0.0008
OverallCD8$^-$FoxP3$^+$PD-L1^{mid}	1.009 (1.0009-1.0172)	0.001
Tumor FoxP3$^+$PD-L1^{mid}	1.0044 (1.0003-1.0084)	0.001
Stroma CD8$^-$FoxP3$^+$PD-1$^-$	1.004 (1.0003-1.0077)	0.0015
Stroma CD8$^-$FoxP3$^+$PD-L1$^+$	1.0031 (1.0004-1.0058)	0.0046
OverallCD8$^-$FoxP3$^+$PD-L1^{low}	1.0223 (1.0013-1.0438)	0.0065
OverallFoxP3$^+$PD-L1^{mid}	1.0065 (1.0006-1.0124)	0.0084
Stroma CD8$^-$FoxP3$^+$	1.0027 (1.0002-1.0052)	0.0171
Tumor CD8$^+$FoxP3$^-$	0.9974 (0.9951-0.9998)	0.0266
OverallCD8$^+$FoxP3$^-$PD-1$^-$	0.9977 (0.9955-0.9999)	0.0324
Stroma CD8$^-$FoxP3$^+$PD-L1^{high}	1.004 (1-1.008)	0.035
Tumor CD8$^+$FoxP3$^-$PD-1$^-$	0.9975 (0.995-1)	0.042
Stroma FoxP3$^+$PD-L1^{mid}	1.0046 (0.9997-1.0095)	0.05
Tumor CD8$^-$FoxP3$^+$PD-1$^-$PD-L1$^-$	1.0071 (0.9985-1.0158)	0.0512
OverallFoxP3$^+$PD-L1^{low}	1.0141 (0.9987-1.0298)	0.0528
Tumor CD8$^+$FoxP3$^-$PD-L1$^+$	0.9977 (0.9951-1.0002)	0.0571
OverallCD8$^+$PD-1$^-$	0.9983 (0.9966-1.0001)	0.0585
Stroma CD8$^+$FoxP3$^-$PD-1$^-$	0.9983 (0.9965-1.0002)	0.0595
Stroma other_PD-L1^{mid}	1.0008 (1-1.0017)	0.061
Tumor CD163$^+$PD-L1^{mid}	1.002 (0.9998-1.0043)	0.0683
Tumor CD8$^+$PD-1$^-$	0.9983 (0.9965-1.0002)	0.0694
Tumor FoxP3$^+$PD-1$^-$PD-L1$^-$	1.0063 (0.9984-1.0143)	0.07
Stroma other_PD-L1^{low}	1.0012 (0.9999-1.0026)	0.0729
OverallCD8$^+$FoxP3$^-$PD-L1^{high}	0.9976 (0.9946-1.0005)	0.0733
Tumor CD8$^+$FoxP3$^+$PD-L1^{low}	1.0364 (0.9922-1.0825)	0.0825
Tumor CD8$^+$FoxP3$^-$PD-L1^{high}	0.9975 (0.9943-1.0007)	0.0883
Tumor CD8$^+$	0.9988 (0.9973-1.0002)	0.094
Stroma CD8$^+$PD-L1^{high}	0.9985 (0.9965-1.0004)	0.0982
Overallother_PD-L1^{mid}	1.0007 (0.9999-1.0014)	0.0993
Stroma CD8$^-$FoxP3$^+$PD-L1^{low}	1.0164 (0.9963-1.0369)	0.0994
Stroma FoxP3$^+$PD-1$^-$	1.0022 (0.9994-1.005)	0.1009
Stroma CD8$^+$FoxP3$^-$PD-L1^{high}	0.9983 (0.9961-1.0005)	0.1071
Stroma CD8$^+$PD-1$^-$	0.9988 (0.9974-1.0003)	0.1099
OverallCD8$^+$FoxP3$^-$	0.9987 (0.9971-1.0004)	0.1169
Tumor CD8$^-$FoxP3$^+$PD-L1$^-$	1.006 (0.9979-1.0142)	0.1229
Tumor CD8$^+$FoxP3$^-$PD-L1^{mid}	0.9909 (0.9791-1.0028)	0.1258
Stroma PD-L1^{mid}	1.0005 (0.9999-1.001)	0.1263
OverallPD-L1^{mid}	1.0005 (0.9999-1.0011)	0.1274
Tumor CD163$^+$PD-L1^{low}	1.0063 (0.9979-1.0147)	0.1398

Stroma CD8^{-}FoxP3^{+}PD-1^{low}	1.0354 (0.9878-1.0853)	0.1407
OverallCD8^{+}PD-L1^{high}	0.9987 (0.9969-1.0005)	0.152
Stroma FoxP3^{+}PD-L1^{+}	1.0014 (0.9994-1.0034)	0.1535
OverallCD8^{+}FoxP3^{-}PD-L1^{+}	0.9989 (0.9972-1.0005)	0.1553
Overallother_PD-L1^{low}	1.0009 (0.9996-1.0021)	0.1567
Tumor CD8^{+}FoxP3^{+}PD-L1^{mid}	1.0206 (0.9909-1.0512)	0.1632
Tumor FoxP3^{+}PD-L1^{-}	1.0051 (0.9976-1.0126)	0.1642
Tumor CD8^{+}PD-1^{-}PD-L1^{-}	0.9949 (0.9875-1.0023)	0.1649
OverallCD8^{+}	0.9992 (0.998-1.0004)	0.1815
Tumor CD8^{+}PD-L1^{mid}	0.9936 (0.9841-1.0032)	0.183
Tumor CD8^{+}PD-L1^{-}	0.9963 (0.9909-1.0018)	0.1839
Tumor CD8^{+}PD-L1^{+}	0.9991 (0.9977-1.0005)	0.1871
Stroma PD-L1^{low}	1.0006 (0.9997-1.0014)	0.1874
Tumor CD8^{+}PD-L1^{low}	0.9918 (0.9793-1.0045)	0.1952
OverallPD-L1^{low}	1.0006 (0.9996-1.0016)	0.2199
Stroma FoxP3^{+}	1.0012 (0.9992-1.0031)	0.2292
Stroma FoxP3^{+}PD-1^{low}	1.0153 (0.9898-1.0416)	0.2354
Tumor CD8^{+}FoxP3^{-}PD-1^{low}	0.9677 (0.9163-1.022)	0.2386
OverallCD8^{+}PD-L1^{+}	0.9993 (0.9981-1.0005)	0.2412
OverallCD8^{-}FoxP3^{+}PD-1^{-}	1.0013 (0.9991-1.0034)	0.2433
Tumor CD8^{-}FoxP3^{+}PD-1^{-}	1.0006 (0.9995-1.0017)	0.2478
Stroma CK^{+}PD-L1^{low}	1.0329 (0.9739-1.0955)	0.2736
OverallCD8^{+}FoxP3^{+}PD-1^{low}	1.0103 (0.9905-1.0305)	0.2967
Tumor CD8^{+}PD-L1^{high}	0.9993 (0.9978-1.0007)	0.3048
OverallCD8^{+}FoxP3^{-}PD-1^{-}PD-L1^{-}	0.9976 (0.9929-1.0023)	0.3064
Tumor CD8^{+}FoxP3^{-}PD-1^{mid}	0.9838 (0.9531-1.0156)	0.3099
Stroma FoxP3^{+}PD-L1^{low}	1.0064 (0.9937-1.0192)	0.3148
OverallCD8^{+}PD-1^{-}PD-L1^{-}	0.9977 (0.9931-1.0023)	0.3178
Stroma CD8^{+}FoxP3^{-}PD-1^{-}PD-L1^{-}	0.9982 (0.9947-1.0018)	0.3196
Stroma CD8^{+}PD-L1^{+}	0.9996 (0.9987-1.0005)	0.3334
Stroma CD8^{+}FoxP3^{-}	0.9996 (0.9986-1.0005)	0.3455
OverallCD8^{+}FoxP3^{-}PD-L1^{-}	0.9984 (0.995-1.0018)	0.3487
Tumor CK^{+}PD-L1^{low}	1.0006 (0.9993-1.0018)	0.358
Tumor CD8^{+}FoxP3^{+}PD-1^{low}	1.0045 (0.9946-1.0145)	0.3593
OverallCD8^{+}PD-L1^{-}	0.9985 (0.9952-1.0018)	0.3641
OverallCD8^{+}FoxP3^{+}PD-L1^{low}	1.0232 (0.9727-1.0763)	0.369
Stroma CD163^{+}PD-L1^{+}	1.0004 (0.9996-1.0012)	0.373
Tumor PD-L1^{low}	1.0004 (0.9995-1.0014)	0.3734
Overallother_PD-L1^{+}	1.0001 (0.9999-1.0003)	0.3768
Stroma CD8^{+}FoxP3^{-}PD-L1^{-}	0.9989 (0.9964-1.0014)	0.38
OverallCD8^{-}FoxP3^{+}PD-L1^{+}	1.0006 (0.9992-1.002)	0.3873
Tumor CD8^{+}FoxP3^{-}PD-1^{+}	0.9973 (0.9909-1.0036)	0.3874
Stroma CD8^{+}FoxP3^{-}PD-L1^{+}	0.9996 (0.9986-1.0005)	0.3886
Stroma other_PD-L1^{+}	1.0001 (0.9999-1.0003)	0.3948
Tumor CD8^{+}FoxP3^{-}PD-L1^{-}	0.9978 (0.9926-1.003)	0.3955
OverallCD163^{+}PD-L1^{-}	0.999 (0.9967-1.0013)	0.4025

Stroma CD8^{+}FoxP3^{+}PD-1^{low}	1.0217 (0.971-1.0751)	0.4037
Stroma CD163^{+}PD-L1^{high}	1.0005 (0.9994-1.0015)	0.4058
Tumor CD8^{+}FoxP3^{-}PD-1^{-}PD-L1^{-}	0.9971 (0.9903-1.004)	0.4107
Stroma FoxP3^{+}PD-L1^{high}	1.0013 (0.9982-1.0043)	0.4153
Stroma CD8^{+}	0.9997 (0.999-1.0004)	0.422
OverallCD8^{+}FoxP3^{+}PD-L1^{mid}	1.0073 (0.9893-1.0257)	0.4229
OverallCK^{+}PD-L1^{high}	0.9995 (0.9984-1.0007)	0.4237
OverallCD8^{-}FoxP3^{+}	1.0006 (0.9992-1.002)	0.4266
OverallCK^{+}	0.9999 (0.9996-1.0002)	0.4445
Stroma CD8^{+}FoxP3^{+}PD-1^{-}	0.9962 (0.9862-1.0062)	0.45
Stroma CD8^{+}FoxP3^{+}PD-L1^{high}	0.9969 (0.9889-1.005)	0.4525
Stroma CK^{+}PD-L1^{high}	0.9813 (0.9338-1.0313)	0.4545
Stroma FoxP3^{+}PD-1^{-}PD-L1^{-}	0.9932 (0.9752-1.0116)	0.4656
OverallFoxP3^{+}PD-1^{-}PD-L1^{-}	0.9923 (0.9717-1.0134)	0.4696
OverallCD163^{+}PD-L1^{low}	1.0024 (0.9958-1.009)	0.4755
Tumor FoxP3^{+}PD-1^{-}	1.0003 (0.9994-1.0012)	0.482
Stroma CD8^{-}FoxP3^{+}PD-1^{+}	1.0025 (0.9955-1.0096)	0.485
Tumor other_PD-L1^{low}	1.0004 (0.9993-1.0015)	0.4917
Tumor CD8^{-}FoxP3^{+}	1.0002 (0.9995-1.001)	0.4919
OverallCD8^{+}FoxP3^{+}PD-1^{mid}	1.0044 (0.9917-1.0172)	0.4946
Tumor CD163^{+}PD-L1^{-}	0.999 (0.9962-1.0019)	0.5006
Tumor CD8^{+}FoxP3^{+}PD-1^{+}	1.001 (0.9981-1.0038)	0.5024
OverallCD8^{-}FoxP3^{+}PD-1^{-}PD-L1^{-}	0.9922 (0.9694-1.0155)	0.5061
OverallCD8^{+}FoxP3^{+}PD-1^{+}	1.0019 (0.9963-1.0075)	0.5082
OverallCD163^{+}PD-L1^{mid}	1.001 (0.9979-1.0042)	0.5146
Tumor CD8^{+}FoxP3^{+}PD-1^{-}PD-L1^{-}	1.0295 (0.9425-1.1245)	0.5151
Tumor CD8^{+}FoxP3^{+}PD-1^{mid}	1.0019 (0.996-1.008)	0.5172
Tumor CD8^{+}FoxP3^{-}PD-1^{high}	0.9975 (0.9899-1.0052)	0.5189
Tumor PD-L1^{mid}	1.0002 (0.9997-1.0007)	0.5255
OverallCK^{+}PD-L1^{-}	0.9999 (0.9995-1.0002)	0.5268
Tumor CK^{+}PD-L1^{high}	0.9998 (0.9993-1.0003)	0.5307
Tumor CD8^{+}FoxP3^{-}PD-L1^{low}	0.9955 (0.9814-1.0098)	0.5314
Stroma CD8^{-}FoxP3^{+}PD-1^{-}PD-L1^{-}	0.9938 (0.9743-1.0138)	0.5408
OverallCD163^{+}PD-L1^{+}	1.0002 (0.9996-1.0007)	0.5416
Stroma CD163^{+}PD-L1^{mid}	1.0008 (0.9983-1.0033)	0.5463
Stroma CD8^{-}FoxP3^{+}PD-1^{mid}	1.0062 (0.9862-1.0265)	0.5464
Stroma PD-L1^{+}	1.0001 (0.9999-1.0002)	0.5475
OverallPD-L1^{+}	1.0001 (0.9999-1.0002)	0.5478
Tumor CD8^{-}FoxP3^{+}PD-L1^{+}	1.0002 (0.9995-1.001)	0.5536
Tumor CD163^{+}PD-L1^{+}	1.0001 (0.9998-1.0004)	0.5626
Tumor PD-L1^{high}	0.9999 (0.9997-1.0002)	0.5702
Stroma CD163^{+}PD-L1^{low}	1.0013 (0.9967-1.006)	0.5708
Stroma CD8^{-}FoxP3^{+}PD-1^{high}	1.0036 (0.9905-1.0169)	0.5909
OverallFoxP3^{+}PD-L1^{-}	0.9963 (0.983-1.0099)	0.5937
OverallFoxP3^{+}PD-1^{-}	1.0005 (0.9987-1.0023)	0.6023
Stroma CD163^{+}PD-L1^{-}	0.9995 (0.9977-1.0014)	0.6027

Stroma FoxP3^{+}PD-1^{mid}	1.0032 (0.9911-1.0155)	0.604
OverallCD163^{+}PD-L1^{high}	1.0002 (0.9995-1.0008)	0.6077
Stroma CK^{+}PD-L1^{mid}	1.0131 (0.9634-1.0653)	0.612
OverallCD8^{-}FoxP3^{+}PD-L1^{-}	0.9961 (0.9812-1.0113)	0.6149
Tumor CD163^{+}	1.0001 (0.9997-1.0004)	0.6262
Tumor CD8^{+}PD-1^{high}	0.9985 (0.9925-1.0046)	0.6282
Stroma CD163^{+}	1.0002 (0.9995-1.0009)	0.6292
Tumor other_PD-L1^{high}	0.9999 (0.9995-1.0003)	0.6365
Stroma FoxP3^{+}PD-L1^{-}	0.9973 (0.9861-1.0086)	0.6375
Tumor CD8^{+}FoxP3^{+}PD-L1^{-}	1.0157 (0.9504-1.0854)	0.6456
Stroma CD8^{+}FoxP3^{+}PD-L1^{low}	1.0071 (0.9768-1.0385)	0.647
OverallCK^{+}PD-L1^{+}	0.9999 (0.9993-1.0004)	0.6508
Stroma FoxP3^{+}PD-1^{+}	1.0008 (0.9974-1.0042)	0.6519
Tumor PD-1^{high}	0.9995 (0.9972-1.0018)	0.656
Tumor CD8^{+}FoxP3^{+}PD-1^{high}	1.0027 (0.9905-1.015)	0.6633
Stroma CD8^{+}FoxP3^{+}PD-L1^{mid}	1.0022 (0.9921-1.0123)	0.6717
Stroma CD8^{-}FoxP3^{+}PD-L1^{-}	0.9974 (0.985-1.0099)	0.6798
OverallFoxP3^{+}PD-L1^{+}	1.0002 (0.9992-1.0012)	0.6952
Stroma CD8^{+}FoxP3^{+}PD-1^{mid}	1.0059 (0.9758-1.0369)	0.705
OverallCD8^{+}FoxP3^{-}PD-1^{low}	0.9962 (0.9764-1.0163)	0.7052
Tumor CD8^{+}FoxP3^{+}	1.0004 (0.9985-1.0023)	0.7066
OverallCD8^{-}FoxP3^{+}PD-L1^{high}	1.0003 (0.9986-1.0021)	0.7116
Tumor CD8^{+}FoxP3^{+}PD-L1^{+}	1.0003 (0.9985-1.0022)	0.717
Tumor other_PD-1^{high}	0.9987 (0.9918-1.0057)	0.7201
Stroma other_PD-1^{mid}	0.9993 (0.9955-1.0031)	0.7247
Tumor FoxP3^{+}	1.0001 (0.9996-1.0006)	0.7251
OverallCD8^{-}FoxP3^{+}PD-1^{low}	1.0031 (0.9857-1.0209)	0.7262
Stroma other_PD-1^{low}	0.9993 (0.9951-1.0034)	0.7268
OverallCD8^{+}FoxP3^{-}PD-L1^{mid}	0.9994 (0.9958-1.0029)	0.7281
OverallFoxP3^{+}	1.0002 (0.9992-1.0012)	0.7319
Overallother_PD-1^{high}	0.9991 (0.994-1.0043)	0.7349
Stroma other_PD-1^{+}	0.9998 (0.9984-1.0011)	0.7358
Overallother_PD-1^{mid}	0.9993 (0.9951-1.0035)	0.7361
Tumor CD163^{+}PD-L1^{high}	1.0001 (0.9997-1.0005)	0.7458
Overallother_PD-1^{+}	0.9998 (0.9983-1.0012)	0.747
OverallCD8^{+}FoxP3^{+}PD-1^{-}PD-L1^{-}	1.0155 (0.9235-1.1166)	0.7502
OverallCD8^{+}FoxP3^{+}PD-1^{-}	0.9989 (0.9923-1.0056)	0.7519
OverallCD163^{+}	1.0001 (0.9995-1.0006)	0.7542
Tumor CD8^{+}PD-1^{+}	0.9995 (0.9963-1.0027)	0.7551
OverallCD8^{+}FoxP3^{+}PD-L1^{-}	1.012 (0.935-1.0954)	0.7672
Stroma other_PD-1^{high}	0.9993 (0.995-1.0037)	0.7674
Stroma CD8^{+}FoxP3^{+}PD-L1^{+}	0.9992 (0.9939-1.0046)	0.7682
Overallother_PD-L1^{high}	1 (0.9998-1.0003)	0.77
Overallother_PD-1^{low}	0.9994 (0.9955-1.0034)	0.7703
Tumor FoxP3^{+}PD-L1^{+}	1.0001 (0.9996-1.0006)	0.776
OverallCD8^{+}FoxP3^{+}PD-1^{high}	1.0022 (0.9868-1.0179)	0.777

Stroma CD8$^+$FoxP3$^+$	0.9993 (0.9941-1.0044)	0.7815
OverallFoxP3$^+$PD-1^{low}	1.0013 (0.9921-1.0105)	0.7854
Stroma FoxP3$^+$PD-1^{high}	1.0007 (0.9951-1.0064)	0.7944
Stroma CD8$^+$PD-1^{low}	0.9988 (0.9896-1.008)	0.7947
OverallPD-1^{mid}	0.9997 (0.9973-1.0021)	0.8004
Tumor other_PD-L1^{mid}	1.0001 (0.9995-1.0007)	0.801
Tumor CD8$^+$FoxP3$^+$PD-L1^{high}	1.0002 (0.9983-1.0022)	0.8103
OverallPD-1$^+$	0.9999 (0.9991-1.0007)	0.8156
Stroma CD8$^+$PD-L1^{mid}	0.9998 (0.9984-1.0013)	0.8176
OverallPD-1^{low}	0.9996 (0.9965-1.0028)	0.8255
OverallCD8$^+$PD-L1^{mid}	0.9997 (0.9967-1.0026)	0.8281
OverallCD8$^-$FoxP3$^+$PD-1$^+$	1.0004 (0.9967-1.0041)	0.8309
OverallPD-1^{high}	0.9998 (0.9982-1.0015)	0.8335
OverallCD8$^-$FoxP3$^+$PD-1^{high}	1.0009 (0.9923-1.0096)	0.8391
OverallCD8$^+$PD-1^{low}	1.0013 (0.9884-1.0144)	0.8427
Stroma CD8$^+$FoxP3$^+$PD-1$^+$	1.0006 (0.9943-1.007)	0.8428
Stroma CD8$^+$FoxP3$^-$PD-1^{low}	0.999 (0.9895-1.0087)	0.8458
Stroma PD-1^{low}	0.9997 (0.9967-1.0027)	0.8461
OverallCD8$^+$FoxP3$^-$PD-1$^+$	0.9998 (0.998-1.0016)	0.8466
OverallCD8$^+$FoxP3$^-$PD-1^{mid}	0.9993 (0.992-1.0067)	0.8563
Tumor CK$^+$PD-L1$^+$	1 (0.9997-1.0003)	0.8668
OverallCD8$^+$FoxP3$^-$PD-1^{high}	0.9998 (0.997-1.0025)	0.8669
OverallCD8$^+$FoxP3$^+$	1.0003 (0.9967-1.0039)	0.8677
Tumor PD-1^{low}	1.0002 (0.9981-1.0023)	0.8691
Stroma other_PD-L1^{high}	1 (0.9998-1.0003)	0.871
OverallCD8$^+$FoxP3$^+$PD-L1$^+$	1.0003 (0.9966-1.0039)	0.8779
OverallCD8$^-$FoxP3$^+$PD-1^{mid}	1.0008 (0.9907-1.0109)	0.8826
OverallCK$^+$PD-L1^{low}	0.9998 (0.9976-1.0021)	0.8838
Stroma CD8$^+$FoxP3$^-$PD-L1^{mid}	0.9999 (0.9984-1.0014)	0.8888
Tumor other_PD-1^{low}	1.0002 (0.9977-1.0027)	0.8921
Tumor CK$^+$	1 (0.9998-1.0002)	0.8929
Stroma CD8$^+$PD-L1^{low}	0.9998 (0.9971-1.0025)	0.9008
Tumor FoxP3$^+$PD-1^{high}	0.9998 (0.996-1.0035)	0.9011
Stroma CK$^+$PD-L1$^+$	1.0014 (0.9787-1.0247)	0.9026
OverallCD8$^+$FoxP3$^-$PD-L1^{low}	0.9997 (0.9941-1.0053)	0.905
Tumor PD-1$^+$	1 (0.9993-1.0007)	0.905
Stroma PD-1^{mid}	0.9999 (0.9982-1.0016)	0.9077
Stroma CD8$^+$PD-1$^-$PD-L1$^-$	1.0002 (0.9973-1.0031)	0.9079
OverallCD8$^+$PD-1^{high}	0.9999 (0.9975-1.0022)	0.9111
Tumor FoxP3$^+$PD-L1^{high}	1 (0.9993-1.0006)	0.9112
OverallFoxP3$^+$PD-1$^+$	1.0001 (0.9979-1.0023)	0.9185
Stroma CD8$^+$FoxP3$^+$PD-L1$^-$	1.0033 (0.9391-1.072)	0.9218
Stroma PD-1$^+$	1 (0.9995-1.0005)	0.9242
Stroma PD-L1^{high}	1 (0.9997-1.0002)	0.9278
Stroma CD8$^+$FoxP3$^+$PD-1$^-$PD-L1$^-$	1.0043 (0.9152-1.102)	0.9281
Tumor CK$^+$PD-L1^{mid}	1 (0.9992-1.0008)	0.931

Tumor CD8$^-$FoxP3$^+$PD-1^{low}	1.0004 (0.9913-1.0096)	0.9328
Tumor FoxP3$^+$PD-1^{low}	1.0002 (0.9955-1.0049)	0.933
OverallCD8$^+$PD-1^{mid}	1.0002 (0.9944-1.0061)	0.9334
Stroma CD8$^+$FoxP3$^-$PD-1^{mid}	0.9999 (0.9964-1.0033)	0.9354
Tumor CD8$^+$PD-1^{mid}	0.9997 (0.992-1.0074)	0.9397
Tumor FoxP3$^+$PD-1^{mid}	0.9999 (0.9971-1.0027)	0.9435
Stroma CD8$^+$FoxP3$^-$PD-1$^+$	1 (0.9991-1.0008)	0.9454
Tumor other_PD-L1$^+$	1 (0.9998-1.0002)	0.9473
OverallFoxP3$^+$PD-1^{high}	1.0002 (0.9943-1.0061)	0.9489
Tumor PD-L1$^+$	1 (0.9998-1.0002)	0.9534
Tumor CD8$^-$FoxP3$^+$PD-1^{high}	0.9998 (0.9945-1.0052)	0.9555
Tumor PD-1^{mid}	0.9999 (0.9982-1.0017)	0.9561
Stroma CD8$^+$PD-1$^+$	1 (0.9992-1.0007)	0.9584
Tumor FoxP3$^+$PD-1$^+$	1 (0.9988-1.0012)	0.959
Stroma PD-1^{high}	1 (0.9991-1.0008)	0.9603
Stroma CD8$^+$PD-1^{mid}	0.9999 (0.9967-1.0031)	0.962
Stroma CD8$^+$FoxP3$^-$PD-1^{high}	1 (0.9988-1.0012)	0.9627
Tumor CD8$^-$FoxP3$^+$PD-L1^{high}	1 (0.9991-1.001)	0.9627
OverallCD8$^+$PD-L1^{low}	0.9999 (0.9949-1.0049)	0.9679
OverallFoxP3$^+$PD-1^{mid}	1.0001 (0.9946-1.0057)	0.9686
Stroma CD8$^+$FoxP3$^+$PD-1^{high}	1.0002 (0.9911-1.0094)	0.9693
Stroma CK$^+$PD-L1$^-$	0.9998 (0.9899-1.0098)	0.9695
OverallCD8$^+$FoxP3$^+$PD-L1^{high}	0.9999 (0.996-1.0039)	0.9722
OverallCD8$^+$PD-1$^+$	1 (0.9985-1.0015)	0.9752
Stroma CD8$^+$PD-1^{high}	1 (0.9989-1.001)	0.9788
Tumor CD8$^-$FoxP3$^+$PD-1^{mid}	0.9999 (0.9947-1.0052)	0.9802
Stroma CD8$^+$FoxP3$^-$PD-L1^{low}	1 (0.9972-1.0027)	0.9808
Tumor CD8$^+$PD-1^{low}	0.9999 (0.9871-1.0128)	0.9821
Tumor CD8$^+$FoxP3$^+$PD-1$^-$	1 (0.9955-1.0044)	0.9824
Tumor CK$^+$PD-L1$^-$	1 (0.9998-1.0002)	0.9859
OverallFoxP3$^+$PD-L1^{high}	1 (0.9988-1.0012)	0.9869
Tumor CD8$^-$FoxP3$^+$PD-1$^+$	1 (0.9979-1.0021)	0.9894
Stroma CK$^+$	1.0001 (0.9907-1.0095)	0.9897
Tumor other_PD-1^{mid}	1 (0.9968-1.0033)	0.9917
Stroma CD8$^+$PD-L1$^-$	1 (0.998-1.002)	0.9946
OverallPD-L1^{high}	1 (0.9998-1.0002)	0.9946
OverallCK$^+$PD-L1^{mid}	1 (0.9985-1.0014)	0.9952
Tumor other_PD-1$^+$	1 (0.9988-1.0012)	0.9964

a.HR = Hazard Ratio, b.CI = Confidence Interval, c. The *p*-values were obtained from the log-rank test.

Table S9. Univariate Cox regression analysis to assess the correlation between the proximity scores of immunocyte subpopulations and immunotherapy-related OS.

**Features (proximity score)**	**HR[a] (95%CI[b])**	***P* value[C]**
CD8^{+}FoxP3^{+}PD-1^{+}	0.638 (0.4714-0.8635)	0.002
CD8^{+}PD-1^{+}	0.8871 (0.8109-0.9705)	0.0071
CD8^{+}FoxP3^{-}PD-1^{+}	0.9058 (0.8389-0.9781)	0.0094
FoxP3^{+}PD-L1^{+}	0.9058 (0.8361-0.9814)	0.0134
CD8^{-}FoxP3^{+}	0.9052 (0.8307-0.9863)	0.0219
CD8^{+}	1.103 (0.9994-1.2175)	0.0484
FoxP3^{+}	1.2641 (0.9499-1.6821)	0.0714
FoxP3^{+}PD-1^{+}	1.0779 (0.9881-1.176)	0.0857
CD8^{+}FoxP3^{+}PD-1^{-}	0.9479 (0.8848-1.0155)	0.1207
PD-1^{+}	0.9309 (0.8465-1.0238)	0.1374
CD163^{+}PD-L1^{-}	0.9252 (0.8166-1.0482)	0.2208
CD8^{+}FoxP3^{-}	1.0654 (0.9624-1.1796)	0.2215
FoxP3^{+}PD-1^{-}PD-L1^{-}	0.9581 (0.8931-1.0279)	0.2304
CD8^{-}FoxP3^{+}PD-1^{+}	0.9611 (0.9002-1.0261)	0.2311
CD8^{-}FoxP3^{+}PD-L1^{-}	0.9585 (0.8915-1.0306)	0.2494
Other_PD-1^{+}	0.9539 (0.8788-1.0354)	0.2567
CD163^{+}PD-L1^{+}	1.0406 (0.9659-1.121)	0.2929
CD8^{+}FoxP3^{+}PD-1^{-}PD-L1^{-}	0.9653 (0.9004-1.035)	0.3149
CD8^{+}FoxP3^{+}PD-L1^{-}	0.9712 (0.9144-1.0314)	0.3361
CD8^{-}FoxP3^{+}PD-1^{-}PD-L1^{-}	0.9668 (0.9007-1.0377)	0.3482
CD8^{+}FoxP3^{+}	0.9689 (0.9066-1.0355)	0.3491
CD8^{+}FoxP3^{+}PD-L1^{+}	0.9703 (0.9101-1.0345)	0.3531
CD163^{+}	1.0548 (0.9386-1.1854)	0.368
CD8^{+}PD-1^{-}	1.0357 (0.95-1.1292)	0.4248
CD8^{+}FoxP3^{-}PD-1^{-}	1.0335 (0.9423-1.1334)	0.4835
Other_PD-L1^{+}	1.0227 (0.9278-1.1273)	0.6503

a.HR = Hazard Ratio, b.CI = Confidence Interval, c. The *p*-values were obtained from the log-rank test.

Table S10. Univariate Cox regression analysis to assess the correlation between the proximity scores of immunocyte subpopulations and immunotherapy-related PFS.

**Features (proximity score)**	**HR[a] (95%CI[b])**	***P* value[C]**
CD8^{+}FoxP3^{+}PD-1^{+}	0.6421 (0.4983-0.8273)	0.0003
CD8^{+}PD-1^{+}	0.8893 (0.8156-0.9696)	0.0065
CD8^{+}FoxP3^{-}PD-1^{+}	0.9058 (0.8397-0.9771)	0.0087
CD8^{+}PD-L1^{-}	1.0782 (0.9812-1.1849)	0.1154
CD8^{-}FoxP3^{+}	0.9434 (0.8757-1.0163)	0.1237
CD8^{+}FoxP3^{+}	0.9552 (0.9002-1.0136)	0.124
CD8^{+}FoxP3^{+}PD-L1^{+}	0.9589 (0.9062-1.0147)	0.1395
CD8^{+}FoxP3^{+}PD-1^{-}	0.9591 (0.9055-1.0159)	0.1487
PD-1^{+}	0.9436 (0.8679-1.0259)	0.1722
CD8^{-}FoxP3^{+}PD-1^{+}	0.9634 (0.9089-1.0213)	0.2068
Other_PD-1^{+}	0.9518 (0.8809-1.0285)	0.2086
CD8^{+}	1.0525 (0.9637-1.1494)	0.2532
FoxP3^{+}PD-L1^{+}	0.9624 (0.8965-1.0332)	0.2885
CD163^{+}PD-L1^{-}	0.957 (0.8646-1.0593)	0.3951
Other_PD-L1^{+}	1.0475 (0.936-1.1722)	0.4172
FoxP3^{+}PD-L1^{-}	0.9609 (0.856-1.0787)	0.4983
CD8^{-}FoxP3^{+}PD-L1^{+}	0.9774 (0.9145-1.0447)	0.5006
CD8^{+}FoxP3^{-}PD-L1^{+}	0.9784 (0.9118-1.0498)	0.5429
CD8^{+}FoxP3^{-}	1.0269 (0.9416-1.1199)	0.5487
FoxP3^{+}	1.0841 (0.8256-1.4234)	0.5559
CD8^{+}FoxP3^{+}PD-L1^{-}	0.9856 (0.9382-1.0354)	0.5631
CD163^{+}	1.0315 (0.9239-1.1516)	0.5805
CD8^{+}FoxP3^{+}PD-1^{-}PD-L1^{-}	0.988 (0.9337-1.0455)	0.6755
CD8^{+}FoxP3^{-}PD-L1^{-}	0.9818 (0.8993-1.0718)	0.6808
CD8^{+}PD-L1^{+}	0.9881 (0.925-1.0554)	0.7211
CD8^{+}PD-1^{-}	1.0134 (0.9399-1.0926)	0.7292

a.HR = Hazard Ratio, b.CI = Confidence Interval, c. The *p*-values were obtained from the log-rank test.
